# Cold-adapted RNA polymerase from *Pseudomonas* phage Njord improves synthesis of therapeutic mRNA

**DOI:** 10.1073/pnas.2606667123

**Published:** 2026-09-02

**Authors:** Dillon B. Nye, Tien-Hao Chen, Jennifer L. Curcuru, Eric J. Wolf, Christa N. Molé, Vidula A. Kunte, Matthew Ryan, Lili Mitchell, Dianne S. Schwarz, Joseph M. Whipple, S. Hong Chan, Ivan R. Corrêa

**Affiliations:** ^a^RNA Division, New England Biolabs, Inc., Beverly, MA 01915

**Keywords:** mRNA therapeutics, in vitro transcription, RNA polymerase, dsRNA

## Abstract

An RNA polymerase identified in the genome of *Pseudomonas* phage Njord offers a promising tool for the synthesis of mRNA and other therapeutic nucleic acids. Originating from a marine microbial ecosystem, Njord RNAP transcribes RNA at high yield even under low temperature conditions. Key properties of the enzyme relevant to mRNA synthesis are presented including transcriptional fidelity, promoter specificity, incorporation of modified nucleotides, and the impurity profile of the RNA. Specific attention is given to the formation of contaminating double-stranded RNA (dsRNA) species. Analysis of transcription reactions shows that DNA-templated promoter-independent transcription is a major source of detectable dsRNA impurities and that Njord RNAP displays a minimal level of this activity. Consistent with the known inflammatory role of dsRNA in synthetic mRNA, transcriptomic analysis of cell culture and a live animal study demonstrates that mRNA synthesized with Njord RNAP elicits only a minimal immune response. This natural enzyme enables efficient mRNA synthesis at ambient temperature and produces transcripts essentially free of dsRNA, offering significant potential to streamline mRNA manufacturing processes.

## Significance

Bacteriophage T7 RNA polymerase (T7 RNAP) has enabled the development of mRNA vaccines and remains central to the manufacture of this therapeutic modality. Here, we report on a natural homolog of T7 RNA polymerase, Njord RNA polymerase, which supports high-yield RNA synthesis at substantially lower temperatures. Njord RNAP generates mRNA with markedly reduced levels of inflammatory RNA contaminants, and comparative analysis of transcription reactions provides insight into the origins of these impurity species. Together, these features position Njord RNA polymerase as a valuable tool for low-temperature RNA synthesis and a promising candidate for improving the performance and scalability of mRNA therapeutics.

The success of messenger RNA (mRNA) vaccines in combating the SARS-CoV-2 pandemic has spurred development of this therapeutic modality. Canonical mRNA, self-amplifying RNA (saRNA), and circular RNA designs have been augmented with sequence design tools, engineered 5′ and 3′ structures, and modified nucleotides to prolong protein expression, evade the innate immune system, and increase efficacy in treating disease (1–9). Ubiquitous in the synthesis and manufacturing of these molecules is the RNA polymerase (RNAP) of bacteriophage T7. Various approaches have been taken to improve this enzyme for in vitro mRNA synthesis. These include engineering of T7 RNAP by directed evolution or computational design, as well as screening natural polymerases derived from the *Autographivirales*, “self-transcribing” phages, for improved properties (10–16). A key metric in the performance of an RNAP for mRNA synthesis is the production of double-stranded RNA (dsRNA) contaminants.

Because of their resemblance to viral genomic RNA species, dsRNA species activate pattern recognition receptors (PRRs) of the mammalian innate immune system (17). These PRRs include toll-like receptor 3 (TLR-3), melanoma differentiation-associated protein 5 (MDA5) and retinoic acid-inducible gene-I (RIG-I), all of which trigger a proinflammatory response. They also include protein kinase R (PKR) and 2′–5′ oligoadenylate synthetases (OASs), which inhibit translation within infected cells. While all these PRRs recognize dsRNA, other structural features are associated with their activation. For instance, RIG-I senses a 5′ triphosphate moiety and is stimulated by influenza RNA (18, 19), whereas OAS1 recognizes a stem loop structure in the 5′ UTR of the SARS-CoV-2 genome (20). Although PRR activation may be avoided by nucleotide modification (21–24), modified dsRNA is nonetheless inflammatory (25). Contaminating dsRNA may be removed from synthetic mRNA by chromatography (26–29), but elimination at the point of synthesis would reduce cost and increase the quality of the drug substance.

Multiple mechanisms by which T7 RNAP produces dsRNA during in vitro transcription (IVT) have been proposed. In one mechanism, the desired RNA folds back on itself and primes the 3′ end to be extended by RNA-dependent RNA polymerase (RdRp) activity of T7 RNAP (30, 31). This RdRp activity is also thought to extend short abortive transcripts when they anneal to the desired full-length mRNA. In a distinct mechanism, T7 RNAP has been shown to initiate transcription at the 3′ terminus of the nontemplate DNA strand in a promoter-independent fashion to produce a fully complementary antisense RNA molecule (13, 32). Each of these processes can contribute to the impurity profile of the drug substance, and distinct dsRNA species may stimulate different components of the innate immune system. Structural determination of dsRNA species present at low levels in synthetic mRNA is challenging. Mechanistic studies using model systems, kinetic analysis of dsRNA production, immuno-northern blots, and RNA sequencing have been applied to understand dsRNA impurities present in synthetic mRNA (31–36). The principal mechanism of dsRNA formation by T7 RNAP remains a matter of debate.

Here, we present an RNAP derived from *Pseudomonas* phage Njord that is a cold-adapted polymerase and produces minimal dsRNA impurities during transcription. Through a comprehensive comparative analysis of T7 and Njord RNA polymerase—integrating biochemical activity, mRNA impurity profiles, and immunological responses in both cell culture and a mouse model—we identify promoter-independent transcription as the predominant source of immunostimulatory dsRNA.

## Results

### Characterization of Njord RNAP for IVT.

**Fig. 1.**
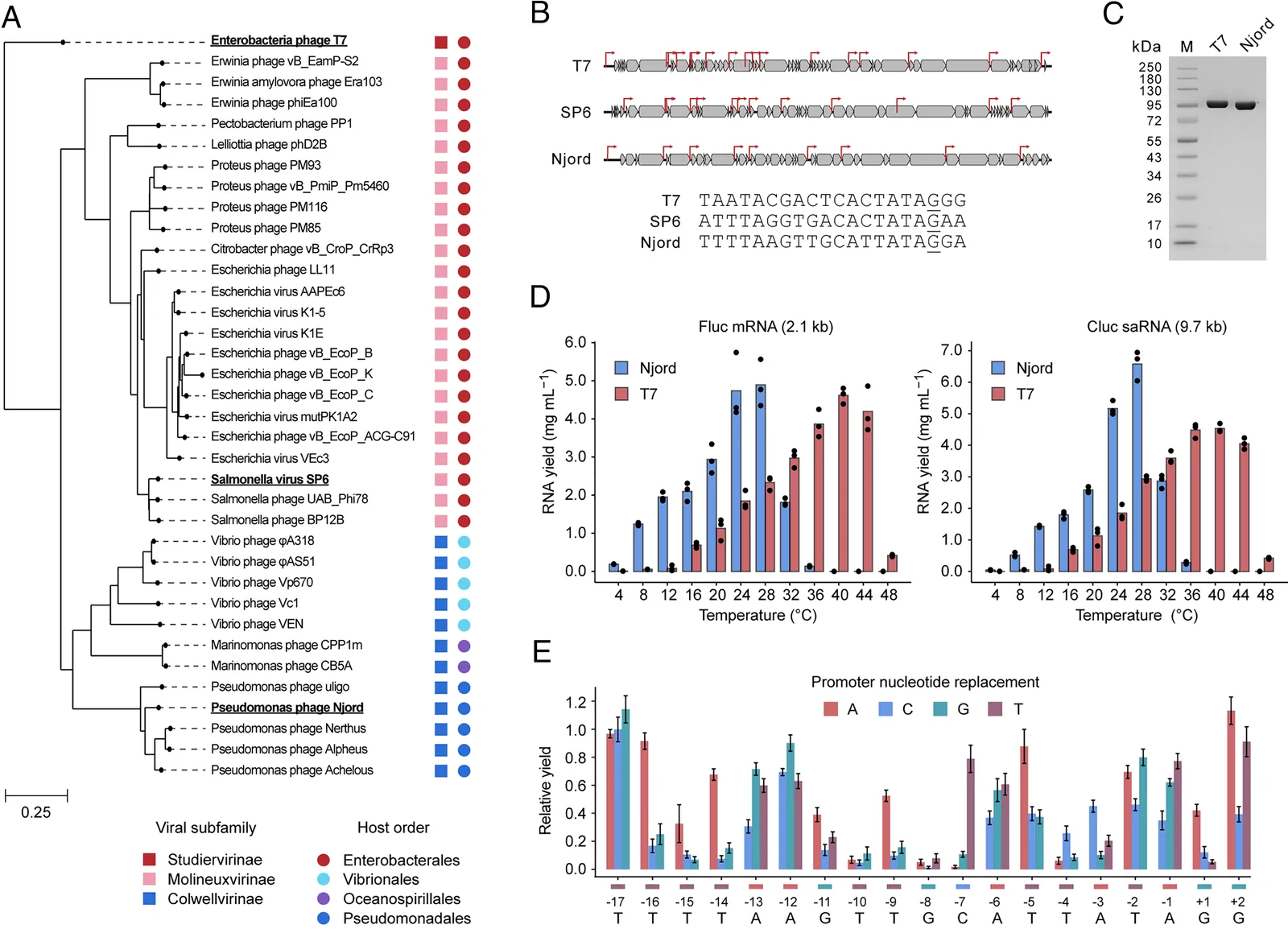
Njord RNAP is a cold-adapted homolog of SP6 RNAP. (*A*) Phylogenetic tree of *Molineuxvirinae* and *Colwellvirinae* bacteriophages. Phage T7 was included and used to root the tree. (*B*) Locations of transcriptional promoters in the genomes of phages T7, SP6, and Njord. Open reading frames are denoted with blocks and promoters are indicated with arrows. Representative promoter sequences are shown with the initiating guanosine underlined. (*C*) SDS-PAGE analysis of T7 RNAP and Njord RNAP preparations. (*D*) RNA yields of Fluc mRNA and saRNA transcripts using T7 and Njord RNAPs at different temperatures. (*E*) Impact of single nucleotide changes to the Njord promoter on RNA yield. Error bars represent SEM (n = 3).

SP6 RNAP was the first polymerase disseminated as an alternative to T7 RNAP for IVT owing to its high transcriptional activity (37). *Salmonella* phage SP6 belongs to the *Molineuxvirinae*, a subfamily in the established taxonomy of viruses (38). Bacteriophages in this subfamily infect different species of *Enterobacteriacae*, while those in the related subfamily *Colwellvirinae* target hosts in the genera *Vibrio*, *Pseudomonas,* and *Marinomonas* (Fig. 1*A*). Isolates of *Colwellvirinae* have been obtained from wastewater, aquaculture tanks, and sea waters. *Vibrio* phages φA318 and φAS51 were isolated from aquaculture waterways and efficiently form plaques when grown at 25 °C (39). *Colwellvirinae* may have evolved to thrive, i.e., carry out rapid lytic infection of the host, in cooler environments than the *Molineuxvirinae*. Given the central role of RNA polymerase in the bacteriophage lytic cycle, we reasoned that *Colwellvirinae* RNAPs may be adapted for efficient transcription at low temperatures. *Pseudomonas* phage Njord RNAP was chosen as a representative *Colwellvirinae* RNAP and tested for its temperature dependence and utility for mRNA synthesis.

To enable IVT with Njord RNAP, its cognate promoter sequence first had to be identified. Searching the phage genome for repeated sequences revealed a motif dispersed throughout intergenic regions (Fig. 1*B*). These repeated sequences were prospectively assigned as promoters and used to build a position-specific weight matrix, identifying nine putative Njord promoters (*SI Appendix*, Table S1). To compare T7 and Njord RNAPs (Fig. 1*C*), linearized plasmids containing the appropriate promoter sequences were used as IVT templates. Two constructs representing a conventional mRNA (2.1 kb) and a saRNA (9.7 kb) were used to assess RNA yield. Transcription was performed at temperatures ranging from 4 to 48 °C. We found that Njord RNAP transcribes optimally at temperatures 10 to 15 °C lower than T7 RNAP, with both yielding similar RNA levels at their optima (Fig. 1*D*). At 12 °C, T7 RNAP is essentially inactive, yet Njord RNAP produces over 1 mg/mL of RNA. Robust RNA synthesis at low temperature indicated that this enzyme is adapted to cooler environments than T7 and SP6 polymerases. This compelling feature of Njord RNAP motivated a detailed evaluation of its potential for therapeutic mRNA synthesis.

Initial transcription reactions with Njord RNAP used a promoter located upstream of the structural genes in analogy to the T7 φ10 promoter. To identify the optimal Njord promoter and gain insight into its promoter sequence specificity, every position in the Njord promoter was systematically replaced (Fig. 1*E*). A preference for guanosine at the +1 position is observed although adenosine may be used as the initiating nucleotide with reduced yield. Most nucleotide replacements reduce the transcriptional yield. Mutation of nucleotides at positions −8 and −10 strongly attenuated transcription, indicating a polymerase–promoter binding mode in common with T7 RNAP (40, 41). Two replacements, T-to-G at position −17 and G-to-A at position +2, marginally increase RNA yield relative to the reference promoter. The resulting sequence corresponds to one of the identified Njord promoters (p4, *SI Appendix*, Table S1) and this promoter was selected for subsequent use in Njord IVT reactions.

**Fig. 2.**
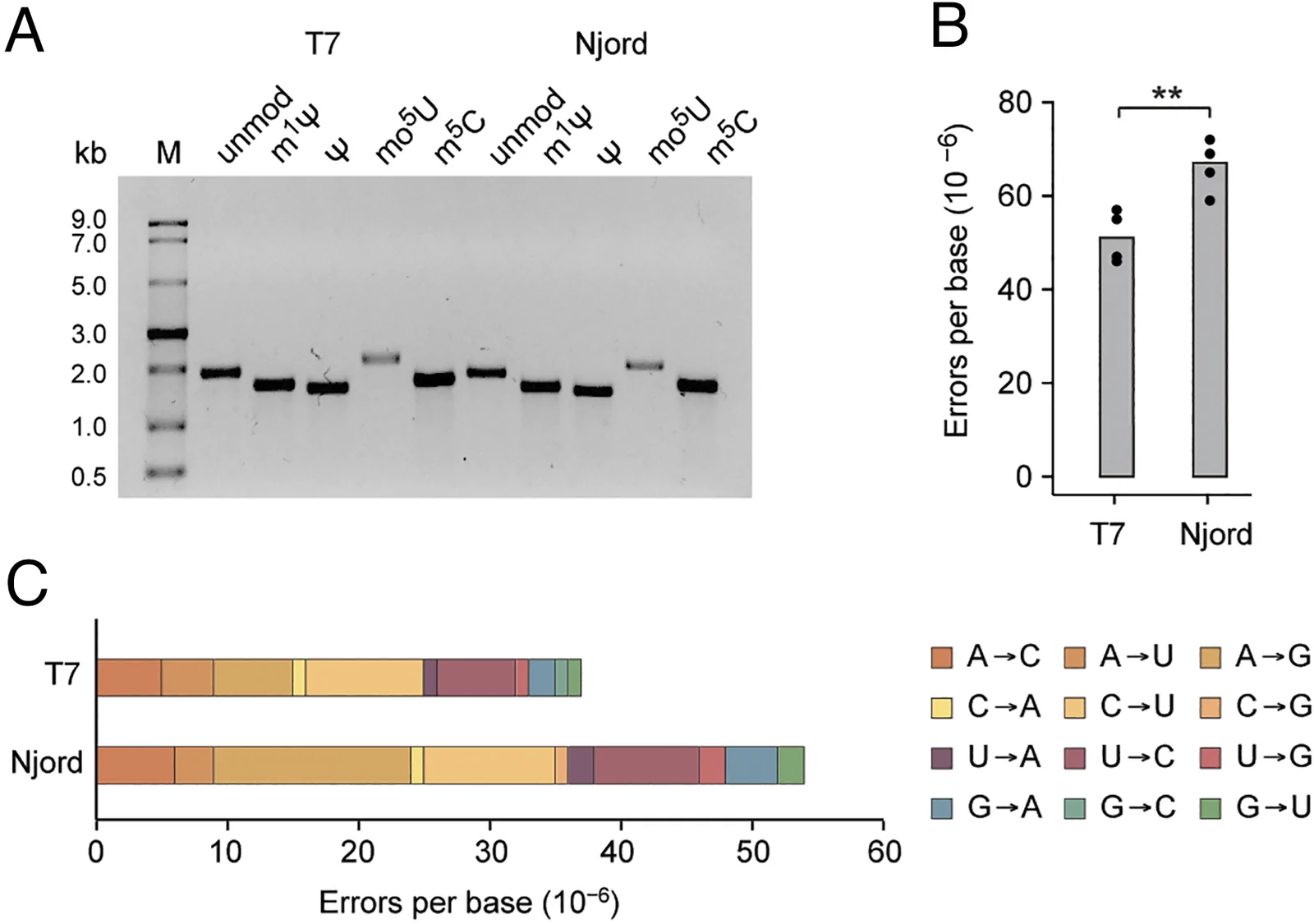
Properties of Njord RNAP relevant for mRNA synthesis. (*A*) Agarose gel electrophoresis analysis of luciferase RNA transcripts with unmodified or modified nucleotides prepared using either T7 or Njord RNAP. (*B*) Comparison of transcriptional fidelities of T7 and Njord RNAPs at 25 °C. Total error rates from four independent experiments using two different template sequences are shown. *P < 0.05; **P < 0.01; ***P < 0.001. (*C*) Frequency of specific substitutions during transcription by T7 and Njord RNAPs. Numerical data on the frequency of specific transversions are available in *SI Appendix*, Tables S3 and S4.

Next, the ability of Njord RNAP to produce accurate mRNA transcripts incorporating modified nucleotides was assessed. Therapeutic mRNAs are commonly produced with modified nucleotides to mitigate undesirable immune stimulation and increase the level of protein expression. Njord RNAP was tested for its ability to incorporate *N*1-methylpseudouridine (m^1^Ψ), pseudouridine (Ψ), 5-methoxyuridine (mo^5^U), and 5-methylcytidine (m^5^C) by substitution of the modified nucleotide for the corresponding unmodified nucleotide triphosphate in the IVT reaction. Modified RNA products displayed shifts in electrophoretic mobility in an agarose gel (Fig. 2*A*) and were essentially identical whether produced using T7 or Njord RNAP.

Transcriptional fidelity denotes the selectivity of the RNA polymerase for the correct nucleotide during transcription. A previously established single-molecule deep sequencing-based assay was used to compare transcriptional fidelities of Njord and T7 RNA polymerases (42). The frequency of nucleotide misincorporation determined for T7 RNAP at 25 °C (51 ± 6 × 10^−6^ errors/base) was comparable to the error rate previously determined at 37 °C (42). The total transcriptional error rate was slightly higher for Njord RNAP (67 ± 8 × 10^−6^ errors/base) (Fig. 2*B*). Although T7 RNAP exhibited a higher propensity for insertion mutations (*SI Appendix*, Table S2), the predominant errors made by both enzymes were substitutions. The apparent elevated frequency of misincorporation by Njord RNAP was attributed to an increase of A|G transversions (Fig. 2*C* and *SI Appendix*, Table S3), suggesting that the active site of Njord RNAP is more accommodating of G/T mismatches resulting from wobble base-pairing. It is likely that these substitution errors can be ameliorated in part by tuning nucleotide triphosphate concentrations according to the desired mRNA sequence (42). With an average misincorporation rate below 1 in 10,000 bases, the observed error frequency is not expected to significantly impact the synthesis of typical mRNA products.

Therapeutic mRNA requires a 5′ cap moiety and Njord RNAP was tested for its ability to incorporate synthetic cap analogs at the start of transcription. When trinucleotides Cap AG or Cap AU were included in the IVT reactions, Njord RNAP produced low yields of partially capped mRNA. In contrast, T7 RNAP efficiently incorporated these cap analogs (*SI Appendix*, Fig. S1 *A*–*C*). Both polymerases were able to incorporate the dinucleotide antireverse cap analog and could produce mRNA that was over 90% capped (*SI Appendix*, Fig. S1 *D*–*F*). Differential reactivities with cap analogs between Njord and T7 prompted an investigation of the effect of the initially transcribed sequence (ITS) on the 5′ mRNA structure and homogeneity (*SI Appendix*, Fig. S2). When the ITS commonly used with T7, GGG, was used with Njord, an additional nontemplated G residue was often added. The 5′ homogeneity of Njord transcripts was highest when the ITS GAA was used. Trinucleotide cap analogs commonly used with T7 RNAP function poorly with Njord RNAP and additional work is needed to enable cotranscriptional capping with this polymerase.

### dsRNA Production by Njord RNAP.

**Fig. 3.**
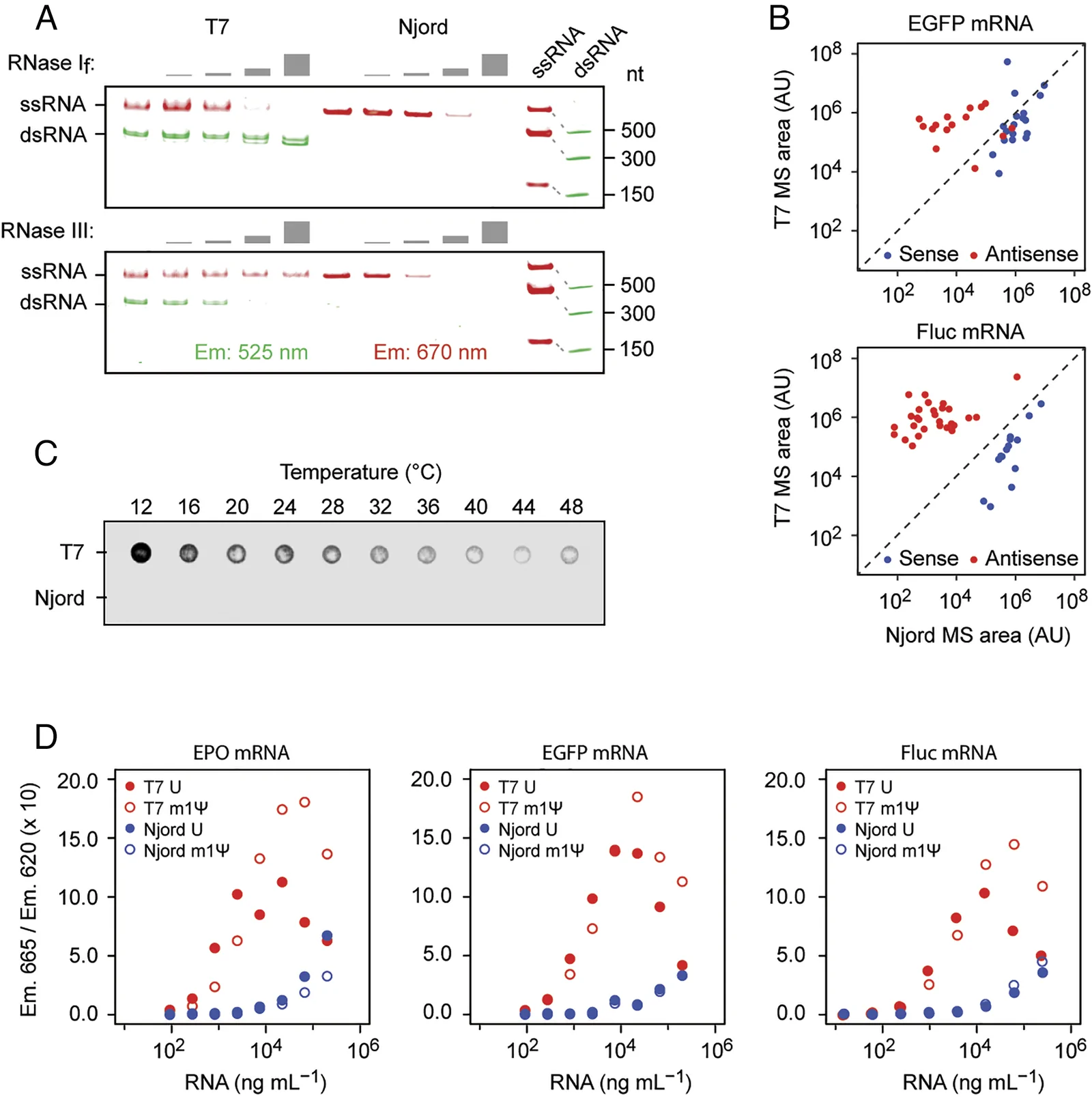
Njord RNAP produces low levels of dsRNA through the promoter-independent mechanism. (*A*) Native PAGE of RNA produced using a DNA template prone to promoter-independent transcription. Digestion of RNA by either RNase I_f_ or RNase III is shown and acridine orange stain was visualized at two wavelengths to discriminate ssRNA and dsRNA. (*B*) Comparison of 5′-triphosphate oligoribonucleotide levels present in mRNA synthesized by either T7 or Njord RNAP. Oligonucleotides were assigned and quantified by UPLC–MS/MS. (*C*) Immunoblot using the J2 antibody to detect dsRNA present in RNA synthesized at different temperatures. (*D*) HTRF assay for detection of dsRNA present in modified and unmodified mRNAs synthesized by either T7 or Njord RNAP.

Having demonstrated that Njord RNAP may be used for ambient temperature synthesis of modified mRNA, we next investigated dsRNA production by this enzyme. Experiments using model substrates were conducted to investigate loopback priming and promoter-independent transcription activities (*SI Appendix*, Fig. S3), and to compare these phenomena between Njord and T7 RNA polymerases. In the first experiment, each RNAP was combined with a fluorescently labeled RNA oligonucleotide and NTPs. The selected RNA has been demonstrated to serve as a substrate for the loopback priming activity of T7 RNAP (31). RNA extension was assessed by capillary electrophoresis. Both T7 and Njord RNAPs showed similar short extension on the RNA oligonucleotide (*SI Appendix*, Fig. S4). As reaction temperatures increased, Njord RNAP activity declined, preventing further comparison. Promoter-independent transcription was assessed in a second experiment, using DNA templates containing either the T7 or Njord promoter and a terminal tetraguanosine tract. This terminal sequence was previously shown to promote dsRNA production through the promoter-independent, DNA-templated mechanism (32). As expected, T7 RNAP produced RNA species that migrate faster than the 300-nt runoff transcript on native PAGE (Fig. 3*A*). The multiple fast-migrating bands were attributed to dsRNA, as evidenced by their degradation upon RNase III treatment, resistance to RNase I_f_ digestion, and ability to intercalate acridine orange dye. Modifying the template upstream of the promoter changed the migration patterns in a predictable fashion (*SI Appendix*, Fig. S5), confirming that the dsRNA arose from promoter-independent activity (13). Njord RNAP generated negligible amounts of these large dsRNA species.

Abortive transcription is common among RNAPs (43) and results in the synthesis of short oligoribonucleotides during promoter escape. These abortive transcripts may, in principle, anneal to full-length mRNA and prime RNAP for extension and synthesis of dsRNA. Mass spectrometry (MS) was employed to assess abortive transcription and identify extended antisense RNA species. To this end, a tandem MS approach was developed to detect 5′ triphosphate species corresponding to either the sense or antisense strand of the expected mRNA. Purified mRNA samples encoding firefly luciferase (Fluc) and enhanced green fluorescent protein (EGFP), synthesized using either T7 or Njord RNAPs, were subjected to MS analysis. Abortive transcripts were produced by both polymerases (Fig. 3*B*), but no evidence for abortive priming of antisense RNA was observed. Instead, 5′ triphosphate antisense oligoribonucleotides mapping to a specific site in the coding sequence of Fluc mRNA were repeatedly identified. This apparent site of transcription initiation did not correspond to an RNAP promoter, but to a serendipitous off-target site for the endonuclease BspQI used to linearize the DNA template (*SI Appendix*, Tables S4 and S5). These species likely arose from abortive promoter-independent transcription and were produced in higher levels by T7 RNAP (Fig. 3*B*). This unbiased search for antisense RNA unexpectedly provided further support for promoter-independent transcription as a differentiator between T7 and Njord RNAPs.

The model substrates employed to assess loopback and promoter-independent transcription were developed for T7 RNAP. To assess dsRNA produced by Njord RNAP in mRNA synthesis, a monoclonal antibody was used. A J2 antibody immunoblot (44) performed on Fluc mRNA confirmed the expected temperature-dependent increase in dsRNA synthesis by T7 RNAP (Fig. 3*C*) (45). In contrast, Njord RNAP produced undetectable levels of dsRNA in this assay. As a complementary quantitative measurement, a J2-based homogeneous time resolved fluorescence (HTRF) assay was employed. To assess the accuracy of the assay and the dependence of the signal on dsRNA length, a set of dsRNA standards was prepared by IVT, followed by annealing and polyacrylamide gel electrophoresis purification (*SI Appendix*, Fig. S6*A*). The assay was accurate for dsRNA species longer than ~200 bp, whereas shorter dsRNA analytes consistently produced lower HTRF signals (*SI Appendix*, Fig. S6*B*). The assay was applied to Fluc, EGFP, and erythropoietin (EPO) mRNAs, synthesized using either T7 or Njord RNAP, with or without m^1^Ψ fully replacing uridine (Fig. 3*D*). Remarkably, across all constructs and regardless of nucleotide modification, Njord produced ~100-fold less dsRNA than T7 RNAP. Compared to cellulose purification of T7 mRNA, Njord RNAP produced lower dsRNA content without compromising RNA yield (*SI Appendix*, Fig. S7). In summary, our findings indicate that dsRNA in IVT mRNA predominantly originates from DNA-templated promoter-independent transcription rather than loopback transcription.

### Immunostimulatory Properties of Synthetic mRNA.

**Fig. 4.**
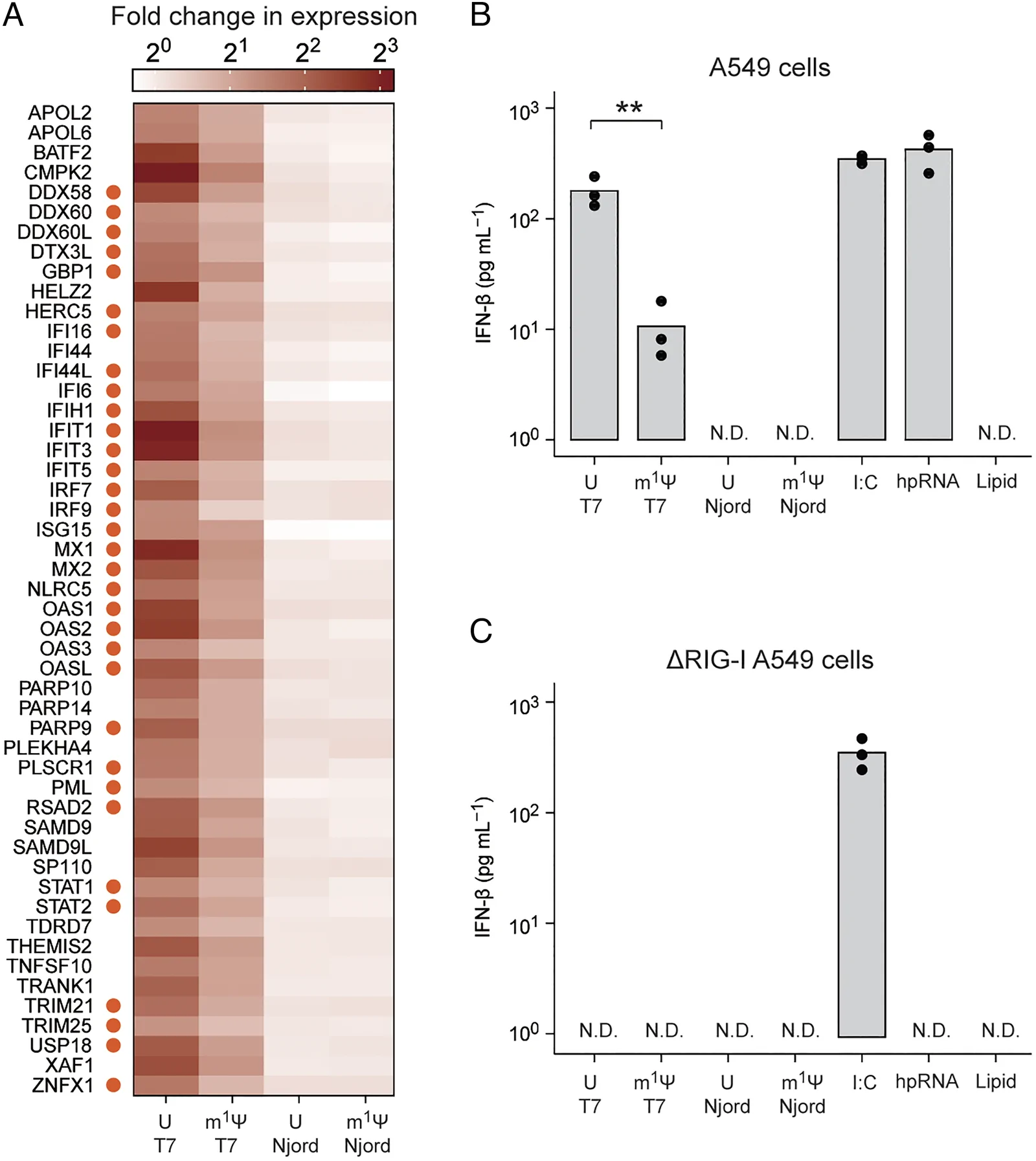
Reduced immune stimulation by mRNA produced with Njord RNAP in A549 human lung cancer cells. (*A*) Transcriptomic analysis of A549 cells following Fluc mRNA transfection. Gene expression changes relative to a mock transfection are shown. Genes involved in viral defense (GO: 0051607) are indicated with a red dot. (*B*) Levels of IFN-β produced by A549 cells in response to Fluc mRNA transfection. (*C*) IFN-β production by RIG-I knockout A549 cells in response to Fluc mRNA transfection. I:C, polyinosinic-polycytidylic acid; hpRNA; RIG-I agonist; Lipid; transfection reagent without RNA; N.D, not detected; *P < 0.05; **P < 0.01; ***P < 0.001.

Lowering the dsRNA burden in IVT mRNA is expected to reduce immune stimulation in eukaryotic cells. Immunostimulatory properties of mRNA generated by Njord RNAP were first tested in A549 cell culture. Fluc mRNA was prepared at the milligram scale using either T7 or Njord RNAP, followed by capping with *Faustovirus* capping enzyme (46). Quality assessments of mRNA integrity, polyadenosine (polyA) tail length and 5′ cap incorporation were consistent across all samples and protein expression was confirmed to be cap-dependent (*SI Appendix*, Figs. S8 and S9). Transcriptome analysis provided a broad view of the cellular response to mRNA transfection. Unmodified and m^1^Ψ-modified mRNA produced with Njord RNAP elicited negligible changes in gene expression, whereas numerous genes were significantly upregulated when T7 RNAP was used (*SI Appendix*, Fig. S10). Many of the differentially expressed genes are annotated with a function in defense response to virus (Fig. 4*A*), and the incorporation of m^1^Ψ in the mRNA attenuated induction of these genes. Consistent with these results, secreted IFNβ levels were significantly reduced by nucleotide modification when T7 RNAP was used and were undetectable with Njord RNAP (Fig. 4*B*). Experiments in RIG-I knockout cells confirmed that this PRR is a primary driver of the IVT mRNA response in the A549 cell line (25) (Fig. 4*C*). These findings were reproduced using multiple lots of linearized plasmid templates and associated Fluc and EGFP mRNA preparations (*SI Appendix*, Fig. S11).

**Fig. 5.**
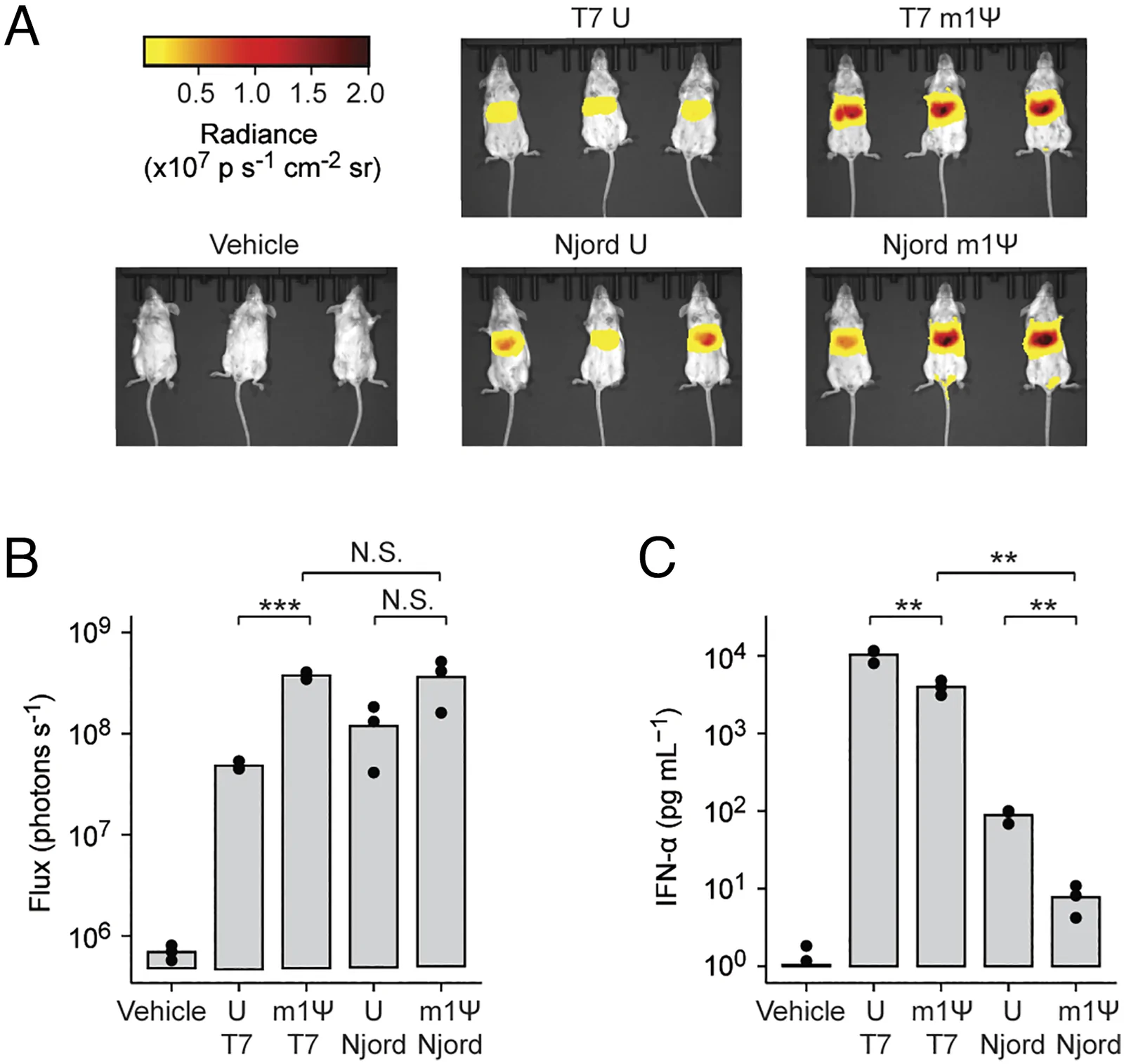
Comparison of immune stimulation and expression levels of mRNA in mice. (*A*) Images of BALB/c mice 6 h after intravenous injection with Fluc mRNA formulated as LNPs. (*B*) Quantification of luciferase expression in mice following transfection. (*C*) Serum IFN-α levels in mice 6 h after treatment with modified and unmodified mRNA-LNPs. N.S., not significant (*P* > 0.05); *P < 0.05; **P < 0.01; ***P < 0.001.

To further validate our findings, we conducted in vivo experiments using modified or unmodified mRNA synthesized by either polymerase. Mice received Fluc mRNA formulated as lipid nanoparticles (LNPs), intravenous injection of which elicited luciferase expression localized to the liver (Fig. 5*A*). Luciferase levels were largely unaffected by the choice of RNAP; however, expression was significantly enhanced by m^1^Ψ incorporation when T7 RNAP was used (Fig. 5*B*). These results reaffirm the benefits of nucleotide modifications in T7 RNAP-based IVT for enhancing mRNA expression in vivo. Unexpectedly, the durability of mRNA expression was not increased by nucleotide modification (47) (*SI Appendix*, Fig. S12), a result that may be attributed to the use of a commercial, nonoptimized LNP reagent. When mice were treated with a higher dose of mRNA-LNP and serum IFN-α levels measured, both RNAP and nucleotide modification emerged as significant factors (Fig. 5*C*). Importantly, m^1^Ψ-modified mRNA synthesized by Njord RNAP at 25 °C was minimally immunostimulatory.

## Discussion

Comparison of the pathways for dsRNA formation by Njord and T7 RNAPs revealed how these impurities influence the immunogenicity of mRNA therapeutics. Both RNAPs readily extend RNA and potentially form loop-back dsRNA structures but are markedly distinct in their ability to engage in promoter-independent transcription. As a result, mRNA transcribed by Njord RNAP contains orders of magnitude less dsRNA species detectable by antibody-based methods. This reduction in dsRNA content correlated with a reduced cytokine response in both cell culture and mouse models. Taken together, our results support DNA-dependent promoter-independent transcription as the source of dsRNA contaminants that activate TLR-3, MDA5, and/or RIG-I and trigger an inflammatory response. Consistent with a recent report (25), nucleotide modification only partially mitigates recognition of dsRNA contaminants. When mRNA synthesized by Njord RNAP was delivered to mice as LNP, incorporation of m^1^Ψ in mRNA-LNPs further reduced serum IFN-α. This effect is likely due to the roles of TLR7 and TLR8 in sensing unmodified endosomal ssRNA (23). Across multiple lots of different mRNA designs and in different biological models, modified mRNA produced using Njord RNAP was nearly immune silent.

In addition to attenuating an inflammatory response, m^1^Ψ enhances expression of mRNA synthesized by T7 RNAP. This effect varies depending on the cell type (48) and has been attributed to masking dsRNA contaminants from recognition by OASs and PKR. Reduced dsRNA content in mRNA made by Njord RNAP could then obviate the benefits of nucleotide modification on protein expression. Our data suggest that this is not the case and that m^1^Ψ-modified mRNA is expressed more effectively regardless of the RNAP used. One potential explanation is that loop-back 3′ extensions form dsRNA structures sufficient to activate OAS1 or PKR. Both T7 and Njord RNAPs produce 3′ extended RNA, which may be difficult to detect with the J2 antibody or remove by chromatography. Alternatively, unmodified ssRNA delivered by an endosome can activate and be degraded by the RNA-binding E3 ubiquitin ligase TRIM25 (49). Additional work is needed to delineate the effects of trace dsRNA impurities and nucleotide modifications on the expression of therapeutic mRNA delivered by LNP.

*Pseudomonas* phage Njord and related viruses of the *Colwellvirinae* originate from marine environments, and Njord RNAP supports efficient RNA synthesis at temperatures of 25 °C and below. Njord RNAP displays a complete loss of activity above 30 °C. This suggests that transcriptional efficiencies of *Autographivirales* RNA polymerases are adapted to the temperature of their environment. Cold adaptation of enzymes has been understood as a trade-off between thermodynamic stability and flexibility to achieve catalytic efficiency (50), although this view has been challenged (51). For T7 RNA polymerase, the effective rate of RNA synthesis in a biomanufacturing context may be limited either by transcription initiation or elongation depending on reaction conditions (34). Adaptations in *Colwellvirinae* RNAPs may tune the kinetics of initiation, elongation, or both, and comparison to warm-adapted *Molineuxvirinae* RNAPs does not reveal obvious sequence determinants for temperature optima. Our work provides a foundation to understand thermal adaptation in this important class of enzymes.

While nearly every aspect of mRNA synthesis—from sequence design to reaction conditions—has been optimized for T7 RNAP, Njord RNAP remains unexplored beyond the scope of this study, highlighting significant opportunities for further development. In particular, Njord RNAP’s ability to permit IVT reactions at ambient or lower temperatures can help preserve the integrity of long nucleic acids (52). We hope that Njord RNAP provides a valuable tool for the research community to further dissect immunological responses to mRNA-based therapies and improve the synthesis of this emerging class of nucleic acid drugs.

## Materials and Methods

Njord RNAP was overexpressed and recombinantly purified using *Escherichia coli* cells. IVT reactions using Njord (25 °C) or T7 (37 °C) RNAPs were performed with or without modified nucleotides or cap analogs on DNA templates corresponding to linearized plasmids or PCR amplicons. Synthesized RNA species were investigated by UV–Vis spectrophotometry or dye-based yield measurements, agarose, acrylamide or capillary gel electrophoresis, long-read sequencing, MS, and dsRNA detection using a J2 dot-blot or a FRET-based assay. Functional Firefly luciferase (Fluc) and EGFP mRNAs were produced using either Njord or T7 RNAPs together with *Faustovirus* capping enzyme. These mRNAs were transfected into WT or ΔRIG-I A549 cells and the cellular responses were measured with fluorescence microscopy, a luciferase reporter assay, an interferon detection assay, and transcriptomic RNA sequencing. Fluc mRNAs were also introduced to BALB/c mice using a commercial LNP reagent. Luciferase expression in live animals was observed by in-vivo imaging and the immunological response to mRNA-LNP measured by quantification of interferon alpha in mouse serum.

Detailed materials and methods are provided in *SI Appendix*.

## Supplementary Material

Appendix 01 (PDF)

## Data Availability

Sequence data have been deposited in Sequence Read Archive (PRJNA1418627) (53). All other data are included in the manuscript and/or *SI Appendix*.
