# Supplementary material for: Cold-adapted RNA polymerase from Pseudomonas phage Njord improves synthesis of therapeutic mRNA
Source: Proc Natl Acad Sci U S A. 2026 Sep 2;123(36):e2606667123. doi: 10.1073/pnas.2606667123 (PMC13552874; doi:10.1073/pnas.2606667123)
Supplement: Supplementary file 1 [file pnas.2606667123.sapp.pdf]

PNAS

Supporting Information for
Cold-adapted RNA polymerase from *Pseudomonas* phage Njord improves synthesis of therapeutic mRNA

Dillon B. Nye[1,*], Tien-Hao Chen[1], Jennifer L. Curcuru[1], Eric J. Wolf[1], Christa N. Molé[1], Vidula A. Kunte[1], Matthew Ryan[1], Lili Mitchell[1], Dianne S. Schwarz[1], Joseph M. Whipple[1], S. Hong Chan[1], and Ivan Reis Corrêa, Jr.[1,*]

[1]RNA Division, New England Biolabs Inc., 240 County Road, Ipswich, MA

*Dillon B. Nye and Ivan Reis Corrêa, Jr.

Email: dnye@neb.com and correa@neb.com

This PDF file includes:

Supporting text
Figures S1 to S12
Tables S1 to S8
Supporting references

Supporting Information Text
Materials and Methods

Materials. Plasmids for Njord RNAP overexpression and for use as templates in IVT reactions were ordered from GenScript (Piscataway, NJ, USA) or prepared by site-directed mutagenesis and are summarized in Table S6. All plasmid sequences were verified by nanopore sequencing using the Rapid Barcoding Kit (Oxford Nanopore Technologies, Oxford, UK, SQK-RBK114.96) and the clone validation assembly workflow. Linearized plasmids were prepared by digestion with BspQI (NEB, R0712) or NdeI (NEB, R0111) and assessed by agarose gel electrophoresis. Oligonucleotides were purchased from IDT (Coralville, IA, USA) and are summarized in Table S7. Information on the RNA species synthesized in this study is provided in Table S8. Unless otherwise noted, all enzymes and reagents were provided by New England Biolabs (NEB).

Njord RNA polymerase promoter identification. Genomes of bacteriophages given the taxonomic assignment of *Colwellvirinae* and *Molineauxvirinae* were collected from the RefSeq database using the webserver search function (1, 2). A phylogenetic tree was built from whole genomes with VICTOR using the d0 formula (3). To identify transcriptional promoters a BLAST alignment was performed using the Njord genome (GCF_003093955.1) as both the query and subject sequence with a cutoff E-value of 0.1 and high scoring pairs were inspected. Promoters identified by BLAST analysis were used to construct a position-specific weight matrix and search the genome for additional instances of the motif with PWMScan (4).

Recombinant purification of Njord RNA polymerase. An expression vector for Njord RNAP (pNjord-01) with an N-terminal hexahistidine tag was designed using the pET28 vector. Competent *E. coli* cells (NEB, C3013) were transformed with the expression plasmid and grown at 37 °C with 220 RPM shaking to an optical density of 0.6 absorbance units. The temperature was reduced to 16 °C, isopropyl β-D-1-thiogalactopyranoside (Gold Biotechnology, Olivette, MO, USA, I2481C) was added to a final concentration of 0.5 mM and protein overexpression allowed to occur overnight. Cultures were centrifuged (3500 rcf, 4 °C, 30 min) and cell pellets were resuspended in Buffer A (50 mM Tris-HCl pH 8.0, 300 mM NaCl, 20 mM imidazole, 10% v/v glycerol) and lysed by sonication. Cellular debris was pelleted by centrifugation (15000 rcf, 4 °C, 45 min) and the clarified lysate was applied to a HisTrap FF column (Cytiva, Marlborough, MA, USA, 17525501). Bound protein was eluted with a step gradient to Buffer B (50 mM Tris-HCl pH 8.0, 0.1 M NaCl, 0.3 M imidazole, 10% v/v glycerol) and fractions were analyzed by sodium dodecyl sulfate-polyacrylamide gel electrophoresis (SDS-PAGE). Fractions containing Njord RNAP were pooled, diluted in Buffer B and applied to a HiTrap Heparin HP column (Cytiva, 17040601). Protein was eluted with a linear gradient to Buffer C (50 mM Tris-HCl pH 8.0, 1.0 M NaCl, 1 mM DTT, 1 mM EDTA, 10% v/v glycerol), diluted in Buffer D (50 mM Tris-HCl pH 8.0, 0.05 M NaCl, 1 mM DTT, 1 mM EDTA, 10% v/v glycerol), applied to a HiTrap Q FF column (Cytiva, 17505301), and finally eluted with a linear gradient to Buffer C. Fractions containing Njord RNAP were pooled and dialyzed against storage buffer (50 mM Tris-HCl pH 8.0, 100 mM NaCl, 0.5 mM TCEP, 0.1 mM EDTA, 50% v/v glycerol). Final purity of Njord RNAP was judged to be >95% by SDS-PAGE and the concentration was measured with a Bradford assay (Bio-Rad, Hercules, CA, USA, 5000205). Purified protein was stored at -20 °C until use. Njord RNAP readily precipitated from solution at low ionic strength or at elevated temperature.

In vitro transcription reactions. Unless otherwise indicated, IVT reactions were performed in IVT buffer (40 mM Tris-HCl pH 7.9, 20 mM $MgCl_2$, 1 mM DTT, 2 mM spermidine) containing 5 mM of each NTP, 1 U/µL murine RNase inhibitor (NEB, M0314), 5 U/mL *E. coli* inorganic pyrophosphatase (NEB, M0361), and 50 ng/µL of linear DNA template. Reactions were pre-incubated at 25 °C (Njord RNAP) or 37 °C (T7 RNAP), and transcription was initiated by addition of RNA polymerase to 10 ng/µL. Following IVT, reactions were treated with 0.1 U/µL DNase I XT (NEB, M0570) at 37 °C for 30 minutes and the RNA was purified using a Monarch RNA Cleanup Kit (NEB, T2050).

Alternatively, the reaction was quenched by addition of equal volume 2X quench buffer (20 mM Tris-HCl pH 8.0, 100 mM EDTA, 0.2% Triton X-100) and analyzed immediately.

RNA yields were measured at different temperatures using plasmid templates for firefly luciferase (pT7-01 and pNjord-02) and self-amplifying RNA (pT7-02 and pNjord-03). These reactions used 20 ng/µL RNAP and were performed in triplicate. Temperature was maintained in a VeritiPro thermal cycler (Thermo Fisher Scientific, Waltham, MA, USA). Reactions were quenched, serially diluted, and combined with Qubit RNA BR buffer (Thermo Fisher Scientific, Q10211). Fluorescence (excitation: 625 nm; emission: 655 nm) was measured using a SpectraMax ID5 (Molecular Devices, San Jose, CA, USA) plate reader. The Qubit kit standards were used to generate a standard curve. To assess incorporation of modified nucleotides, plasmid templates for *Cypridina* luciferase (pT7-03 and pNjord-04) were used. *N*1-methylpseudouridine (NEB, N0431), pseudouridine (NEB, N0433), 5-methoxyuridine (NEB, N0434), and 5-methylcytidine (NEB, N0432) were used in place of the corresponding nucleotide. RNA was purified and quantified by Nanodrop (Thermo Fisher Scientific), then denatured by heating to 70 °C for 5 minutes in RNA Loading Dye (NEB, B0363) and visualized by agarose gel electrophoresis and an ethidium bromide stain.

Njord RNAP promoter optimization. Polymerase chain reaction (PCR) primers were designed to include the 19 nt Njord transcriptional promoter or all 57 possible single-nucleotide substitutions (dOligo01-59). PCR was carried out using Q5 High Fidelity 2X Master Mix (NEB, M0492) using plasmid pT7-03 as template. PCR amplicons were purified using a KingFisher Flex (Thermo Fisher Scientific), assessed with the TapeStation High Sensitivity D5000 kit (Agilent, Lexington, MA, USA, 5067-5593) and quantified with a Nanodrop 8000 (Thermo Fisher Scientific). IVT reactions were performed in triplicate using 10 ng/µL of template at 25 °C for 60 minutes followed by quenching of the reaction, serial dilution and RNA quantification.

RNAP transcriptional fidelity. An assay developed for polymerase fidelity measurement with a Pacific Biosciences (Menlo Park, CA, USA) sequencing platform was applied to Njord and T7 RNAP at 25 °C. These reactions used 15 ng/µL of a linearized plasmid template designed to include all trinucleotide sequences (pT7-04, pT7-05, pNjord-05, and pNjord-06). To achieve sufficient RNA yield at 25 °C, T7 RNAP reactions were performed with 10 mM NTPs and incubated for 2 hours. Synthesis of cDNA, Pacific Biosciences SMRTBell library preparation, sequencing on a Sequel system, and data analysis were performed as described previously (5). Briefly, 10 µg RNA was used with the ProtoScript II cDNA synthesis kit (NEB, E6560) and dOligo60. Following RNase H digestion (NEB, M0297) and spin column purification (NEB, T1030), the same cDNA synthesis kit was used with dOligo61. The cDNA was then purified, end-repaired using NEBNext End Repair Module (NEB, E6050), purified again, and ligated to barcoded adaptors (Pacific Biosciences, 100-466-000) using T4 DNA ligase (NEB, M0202). Unligated DNA and adaptors were removed using Exo III (NEB, M0206) and Exo VII (NEB, M0379). The DNA was repaired with PreCR repair mix (NEB, M0309), purified using AMPure PB Beads (Pacific Biosciences, 100-265-900) and input to the Sequel II binding kit 2.1 (Pacific Biosciences, 101-820-500) for sequencing on a Sequel II system. Data were analyzed as described previously (5).

Assays for dsRNA formation. To assay loopback priming activity, a 5′ fluorescein-modified RNA oligo (rOligo01) was used. Labeled RNA (100 nM) was combined with IVT buffer and 5 mM of each NTP in a volume of 19 µL and pre-incubated for 10 minutes at the specified temperature before addition of 400 ng of T7 or Njord RNAP. For the 0-minute time points, 1 µL water was added in place of polymerase. At the indicated time points reactions were quenched by addition of equal volume 2X quench buffer (20 mM Tris-HCl pH 8.0, 100 mM EDTA, 2% SDS). Reactions were performed in triplicate, diluted 10-fold in water and analyzed by an Applied Biosystems 3730xl (Thermo Fisher Scientific) capillary electrophoresis instrument (6).

To assay DNA-terminus initiated transcription, IVT templates designed to produce unstructured RNA were generated by PCR (dOligo62-64) using plasmid pT7-06. Forward PCR primers installed

the Njord or T7 RNAP promoter and the reverse primer contained a sequence known to promote DNA-terminus initiated transcription. Purified PCR amplicons were used as input for IVT at 30 °C for both RNAPs. RNA was purified, digested by either RNAse III with 20 mM $MnCl_2$ (NEB, M0245) or by RNAse I_f (NEB, M0243) and run on a 6% polyacrylamide TBE gel (Thermo Fisher Scientific, EC6265). Gels were stained with 4.3 µM acridine orange dye (Thermo Fisher Scientific, A1301) and visualized on a Typhoon scanner (Cytiva). Fluorescence images were collected with 488 nm laser excitation and emission filters for either ssRNA (670 nm) or dsRNA (525 nm), then processed and overlaid using Adobe Photoshop. In a complementary experiment, four IVT templates were generated by PCR (dOligo65-67) using plasmids pNjord-04 or pT7-03. These IVT templates contained a region of variable length upstream of the RNAP promoter. IVT reactions using these amplicons were performed at either 37 °C with T7 RNAP or 25 °C with Njord RNAP. Purified RNA was digested by RNAse III then denatured in RNA Loading Dye, run on a 1% agarose gel and stained with SYBR Gold (Thermo Fisher Scientific, S11494).

The dsRNA content in IVT mRNA was measured qualitatively with a J2 immunoblot. Fluc RNA produced using either Njord or T7 RNAP and the appropriate template (pT7-01 or pNjord-02) was purified and quantified using a Nanodrop. For reactions which produced at least 500 ng of RNA, RNA (500 ng) was spotted onto a positively charged nylon membrane (Sigma-Aldrich, St. Louis, MO, USA, WHA10416294) using a 96-well silicone gasket (Bio-Rad, 1706546) taped securely over the membrane and a layer of Whatman paper. Membranes were UV cross-linked (254 nm, 120 mJ/cm^2) and blocked in 5% (w/v) nonfat dried milk in TBS-T buffer (20 mM Tris, pH 7.5, 150 mM NaCl, 0.1% v/v Tween-20). For the detection of dsRNA, the membranes were incubated with a 5,000-fold dilution of J2 anti-dsRNA antibody (SCICONS, Newark, CA, USA, 10010200) at 4°C overnight. The blots were probed with a 10,000-fold dilution of IR Dye-800 conjugated secondary antibodies (Cell Signaling Technologies, Beverly, MA, USA, 5257) and visualized on an Odyssey M Imager (LICORbio, Lincoln, NE, USA). A dsRNA ladder (NEB, N0363) was used as a positive control and a ssRNA ladder (NEB, N0362) was used as a negative control.

A homogeneous time resolved fluorescence (HTRF) dsRNA-detection kit (Revvity, Waltham, MA, USA, 64DSRNAPEG) was also used to measure dsRNA content in IVT RNA. To evaluate the assay, polyadenosine (Sigma-Aldrich, P9403) and a panel of dsRNA standards were used. To prepare a short dsRNA standard, complementary RNA oligos (rOligo02 and rOligo03) were annealed. To prepare the other dsRNA standards, IVT templates were generated by PCR using primers dOligo68-83 and plasmid pT7-02. Njord RNAP was used with these templates to produce complementary strands of RNA which were spin column purified, quantified, and annealed. Annealed dsRNA standards were purified by native PAGE and quantified (7) using a Nanodrop 8000 spectrophotometer. Purified IVT RNA or dsRNA standards were serially diluted in nuclease-free water in a 96-well plate alongside the HTRF manufacturer dsRNA standard. Samples were diluted 10-fold into the kit lysis buffer, and then 10 µL was combined with 10 µL of a 1:1 donor:acceptor antibody mix. HTRF was measured using a SpectraMax ID5 plate reader equipped with a TRF Enhancement Module (Molecular Devices) according to the manufacturer's instructions with an interval delay of 10 µs and an integration time of 200 µs.

Cellulose resin (Sigma-Aldrich, C6288) was used to purify dsRNA from mRNA prepared with T7 RNAP. Cellulose (2 g) was suspended in 10 mL of Buffer CB (10 mM HEPES pH 7.3, 0.1 mM EDTA, 125 mM NaCl, 16% v/v ethanol). The cellulose slurry (0.5 mL) was centrifuged (13000 rcf) in a 0.22 µm filter spin column (Corning Life Sciences, Corning, NY, USA, 8160), resuspended in the same buffer then centrifuged again. Luciferase mRNA was transcribed using either T7 or Njord RNAPs with the appropriate templates (pT7-01 and pNjord-02) at a volume of 50 µL, purified by spin column (NEB, T2050), eluted in 225 µL nuclease-free H_2O and yield was measured by Nanodrop. An equal volume of 2x Buffer CB was added and the buffered RNA solution was used to resuspend the cellulose resin. Following a 30-minute incubation with vigorous shaking, purified RNA was eluted by centrifugation. Purified RNA was precipitated overnight at 4 °C by addition of 50 µL of 3 M ammonium acetate pH 5.5 and then 500 µL of ice-cold isopropyl alcohol. Yield

following purification was measured by Nanodrop and the HTRF assay was used to assess dsRNA content.

Preparation of mRNA for cell culture and in vivo experiments. In vitro transcription of EPO, EGFP and Fluc mRNAs was performed using the appropriate templates (pT7-01, pT7-07, pT7-08, pNjord-02, pNjord-07 and pNjord-08) with either 5 mM UTP or 5 mM m1ΨTP, 15 mM $MgCl_2$ (Fluc) or 20 mM $MgCl_2$ (EPO and EGFP), and 10 ng/µL T7 or Njord RNAP at a scale of 200 µL to produce 0.7 – 1.0 mg RNA. RNA was purified by spin columns (NEB, T2050). To install the 5′ cap, purified RNA (200 µg) was combined in capping buffer (50 mM Tris-HCl pH 8, 5 mM KCl, 1 mM $MgCl_2$, 1 mM DTT, 0.5 mM GTP, 0.2 mM S-adenosyl methionine) together with 200 U Faustovirus Capping Enzyme (NEB, M2081) and 900 U Vaccinia 2'-O-Methyltransferase (NEB, M0366) and incubated at 37°C for 60 minutes. Following this reaction, mRNA was again purified using a spin column.

Mass spectrometry analysis of mRNA 5′ cap incorporation, 5′ homogeneity and 3′ polyA tail length. Co-transcriptional capping IVT reactions were performed under standard IVT conditions with 5 mM each NTP and 5 mM trinucleotide cap analog (CapAG or CapAU, Figure S1A). Trinucleotide cap analogs were purchased from Northern RNA (Calgary, CN). IVT templates were designed to encode saRNA with initiating nucleotides AG or AU corresponding to the cap analog (pT7-09, pT7-10, pNjord-09 and pNjord-10). Alternatively, the dinucleotide anti-reverse cap analog (NEB, S1411) was used at varied concentrations with an appropriate saRNA plasmid template (pT7-02 and pNjord-03) and 2 mM of each NTP. Co-transcriptionally capped RNA was purified using RNAClean XP beads (Beckman Coulter, Brea, CA, USA) and the concentration was measured with a Nanodrop 8000 instrument.

IVT templates encoding mRNA initiating with the trinucleotides GGG, GAA, or GAG were prepared by PCR using appropriate primers (dOligo84 – dOligo90) and plasmid template (pT7-01 or pNjord-02). Transcription was performed under standard conditions. The 5′ RNA homogeneity was assessed by mass spectrometry and compared between T7 and Njord RNAPs.

Measurements of 5′ cap incorporation and 5′ RNA homogeneity were performed using mass spectrometry as previously described (8). Purified RNA was protected at the 5′ end with a complementary biotinylated DNA oligo. For measurement of co-transcriptional capping using saRNA constructs, DNA oligos appropriate for the 5′ sequence (dOligo91 – dOligo93) were used. For measurement of 5′ homogeneity and validation of enzymatic capping of Fluc and EGFP mRNAs, dOligo94 was used. RNA (up to 15 pmole) and protecting oligonucleotide (30 pmole) were combined, heated to 80 °C for 2 minutes then gradually cooled to 20 °C. NEBuffer r1.1 was added to a final 1x concentration (10 mM Bis-Tris-Propane-HCl pH 7, 10 mM $MgCl_2$, 100 µg/mL recombinant BSA) and human RNase IV (5 U, NEB, M1284) was used to digest the RNA at 37 °C for 1 hour and then inactivated by addition of 50 U murine RNase inhibitor. The RNA/DNA heteroduplex was purified with streptavidin beads (NEB, S1420) and eluted by heating the beads in water to 80 °C for 5 minutes. The resultant purified heteroduplex was characterized by ultrahigh performance liquid chromatography mass spectrometry (UHPLC-MS/MS). Briefly, oligonucleotides were separated on a Vanquish UHPLC (Thermo Fisher Scientific) with a Waters ACQUITY Premier Oligonucleotide C18 Column (Waters Corporation, Milford, MA, USA) (2.1 × 100 mm, 1.7 µm). A 23-minute gradient of buffer A (1% hexafluoroisopropanol (HFIP), 0.1% N,N-diisopropylethylamine (DIEA), 1 µM EDTA), and 7–55% buffer B (90% methanol, 10% water, 0.075% HFIP, 0.0375% DIEA, 1 µM EDTA) at a flow rate of 300 µL/min at 70°C was used for separation. Intact oligonucleotide mass spectra were collected on an Orbitrap Eclipse Fusion Mass Spectrometer (Thermo Fisher Scientific) at a resolution of 120K. Accurate mass deconvolution was performed utilizing BioPharma Finder 5.1 (Thermo Fisher Scientific) and annotated with an in-house R script.

Lengths of polyA tails in Fluc and EGFP mRNA were also assessed by UPLC-MS/MS. Purified RNA (15 pmole) was heat denatured at 80 °C for 2 minutes then immediately placed on ice. Buffer was added to a final 1x concentration (50 mM Tris-HCl pH 7.5, 2 mM EDTA) and RNase T1 (500

U, Thermo Fisher Scientific, EN0541) was added to digest the RNA at 37 °C for 60 minutes. Following digestion, 50 U murine RNase inhibitor was added along with $LiCl_2$ to a final concentration of 250 mM then the mixture was combined with pre-washed Oligo dT_{25} beads (NEB, S1419) and incubated at room temperature for 20 minutes. The beads were washed once with Buffer T1 (20 mM Tris-HCl pH 7.5, 50 mM $LiCl_2$, 10 mM EDTA), twice with Buffer T2 (20 mM Tris-HCl pH 7.5, 500 mM $LiCl_2$, 10 mM EDTA), then again with Buffer T1. Purified polyA tails were eluted in 30 µL nuclease-free H_2O by heating to 65 °C then subjected to UHPLC-MS/MS analysis as described for 5′ cap incorporation and 5′ RNA homogeneity measurements.

Capillary gel electrophoresis of RNA. Capillary gel electrophoresis of RNA was performed on a BioPhase8800 instrument (AB SCIEX, Framingham, MA, USA) using laser-induced fluorescence detection (excitation: 488 nm; emission: 520 nm). Experiments were performed using the RNA 9000 Purity and Integrity kit (AB SCIEX, C48231) with the SYBR Green II RNA Gel Stain and 50 µm i.d. × 30 cm bare-fused silica capillaries (AB SCIEX, A55625). Prior to analysis, RNA samples were heated to 70°C for 5 min in the kit sample buffer and immediately placed on ice for at least 5 minutes. The separation method was performed with an electrokinetic injection of the samples using reverse polarity mode at 6.0 kV constant for 22 minutes and 8 Hz data collection rate. The capillary temperature was 30°C. Raw data were exported as plain text and analyzed using an in-house Python script. RNA integrity was calculated by curve fitting a Gaussian function to the peak of interest.

Mass spectrometry analysis of oligoribonucleotides present in IVT mRNA. Uncapped EGFP and Fluc mRNA products (12.5 µg) purified by spin column were directly subjected to UHPLC-MS/MS analysis without further treatment. Oligoribonucleotides present in each mRNA preparation were separated on a Vanquish UHPLC (Thermo Fisher Scientific) with a Waters ACQUITY Premier Oligonucleotide C18 Column (2.1 × 100 mm, 1.7 µm). A 23-minute gradient of buffer A (1% HFIP, 0.1% DIEA, 1 µM EDTA), and 7–35% buffer B (90% methanol, 10% water, 0.075% HFIP, 0.0375% DIEA, 1 µM EDTA) at a flow rate of 300 µL/min at 70°C was applied. Tandem mass spectra were acquired on an Orbitrap Eclipse Tribrid (Thermo Fisher Scientific) mass spectrometer in data dependent MS^2 mode. Intact oligonucleotide MS^1 spectra were acquired at a resolution of 120K. Data dependent MS^2 fragmentation spectra were acquired with a stepped normalized collision energy of 22, 24, 26% at a resolution of 60K. Oligoribonucleotides were identified utilizing BioPharma Finder 5.1 in "Oligonucleotide Mode" using the expected sense and antisense sequences of the mRNA. Oligoribonucleotides assigned with at least 90% confidence and Δ ppm less than 5 ppm are reported in Tables S4 and S5.

Transfection of mRNA in cell culture. A549 (CCL-185) cells from American Type Culture Collection were maintained at 5% CO_2 and 37 °C in Dulbecco's Modified Eagle's Medium (Thermo Fisher Scientific, 10569) supplemented with 10% v/v heat-inactivated fetal bovine serum (Sigma-Aldrich, F9665), and 1% v/v penicillin/streptomycin (Thermo Fisher Scientific, 15140148). A549-Dual (Invivogen, San Diego, CA, a549d) and A549-Dual KO-Rig-I cells (Invivogen, a549d-korigi) were maintained as above but were additionally supplemented with 100 µg/ml Normocin (Invivogen, ant-nr), 10 µg/ml blasticidin (Invivogen, ant-bl), and 100 µg/ml Zeocin (Invivogen, ant-zn).

Cells were seeded in a 96-well plate, cultured to 80% confluency, and transfected with mRNA (200 ng) using jetMESSENGER (Sartorius, Göttingen, DE, 101000005) according to the manufacturer's instructions. Polyinosinic-polycytidylic acid (Invivogen, tlrl-picw), a 5′ segment of influenza genomic RNA (3p-hpRNA) (Invivogen, tlrl-hprna), mo^5U-modified Fluc (TriLink, L-7202) and mo^5U-modified EGFP (TriLink, L-7201) were used as controls, along with mock transfections in which no RNA was used. To control for cell viability and transfection efficiency when measuring expression of test mRNA, cells were co-transfected with 5moU-modified mCherry mRNA (TriLink, L-7203) or Renilla luciferase mRNA (TriLink, L-7204) as appropriate. EGFP expression was observed by imaging A549 cells 24 hours after transfection using an EVOS M7000 imaging system (Thermo Fisher

Scientific). Firefly and Renilla luciferase expression levels were measured using the Dual-Luciferase Reporter Assay System (Promega, Madison, WI, USA, E1910) and a SpectraMax iD5 plate reader equipped with reagent injectors. Secreted interferon beta was measured in cell culture supernatants using a high sensitivity human IFN-beta ELISA kit (PBL Assay Science, Piscataway, NJ, USA, 41435).

Transcriptomic analysis of cellular response to transfection. A549-Dual cells were transfected with Fluc mRNA as described above. Two biological replicates were performed for each condition including a mock transfection using water in place of RNA. Cells were harvested five hours after transfection and RNA was purified using a Monarch Spin RNA Isolation Kit (NEB, T2110). Total RNA (1 μg) was subjected to ribosomal RNA depletion using NEBNext rRNA Depletion Kit v2 (Human/Mouse/Rat) (NEB, E7400). The rRNA-depleted RNA was used for library preparation with NEBNext Ultra II Directional RNA Library Prep Kit for Illumina (NEB, E7760) and NEBNext Multiplex Oligos for Illumina (NEB, E6440). DNA libraries were evaluated and quantified using High Sensitivity D1000 ScreenTape Assay (Agilent, 5067), then pooled to a final concentration of 1 nM. Paired-end sequencing was performed on a NovaSeq 6000 platform (Illumina, San Diego, CA, USA) with the paired-end read length of 100 bp. The raw sequence files were pre-processed to remove adaptor sequence and low-quality reads using FASTX-Toolkit (9). Trimmed sequences were mapped to the human reference genome GRCh38 provided by ENSEMBL using RNA STAR (10). Uniquely mapped transcripts were counted with featureCounts (11) with gene ontologies assigned according to the hg38 reference assembly. Differentially expressed genes relative to no-RNA transfected A549 cells were determined using DESeq2 package version 1.16.1 (12) incorporating both biological replicates.

Live animal study with mRNA-LNPs. Animal experiments were performed at Charles River Laboratories (CRL, Wilmington, MA, USA) and reviewed by their institutional animal care and use committee according to the guidelines of the USA National Research Council. BALB/c mice were used for low-dose and high-dose arms of a study on expression and immunogenicity of mRNA preparations. Three mice were used for each treatment at low and high dosages. All reagents and materials used were provided by CRL except where noted.

Test articles were prepared as LNPs by complexation of Fluc mRNA with Invivofectamine 3.0 (Thermo Fisher Scientific, IVF3005). The mRNA (1 mg/mL) was combined with Complexation Buffer in a 1 to 1 ratio to 80 μL and to this mixture was added 80 μL of the Invivofectamine Reagent, according to the manufacturer's instructions. This solution was thoroughly vortexed and incubated at 50 °C for 30 minutes then diluted to a final volume of 800 μL in Dulbecco's phosphate buffered saline (DPBS). This mRNA-LNP solution (0.05 mg/mL mRNA) was further diluted 10-fold in DPBS and used at either concentration without further purification or analysis. A mock treatment was prepared by substituting water for the mRNA prior to LNP complexation.

To quantify and localize luciferase expression, mRNA-LNPs were introduced to mice via dorsal vein injection at a dosage of 50 mg per kg body weight. Six hours after treatment, mice were further treated with luciferin-D (150 mg/kg) via dorsal vein injection, and within 15 minutes were imaged using an IVIS 200 Spectrum (Revvity) imager. Luciferin injection and imaging were repeated at 24, 48 and 72 hours following mRNA-LNP treatment. Images were processed and analyzed using the Living Image (Revvity) software. In the high-dose arm of the study, mice were treated with mRNA-LNPs at 500 mg per kg body weight. After 6 hours, mice were sacrificed and serum was collected. Interferon alpha (IFN-α) levels were measured in processed serum using the Mouse IFN-Alpha All Subtype High Sensitivity ELISA kit (PBL Assay Science, 42115) and a SpectraMax iD5 plate reader.

Statistical analysis. Statistical significance between two groups was determined using the two-tailed Student's *t*-test implemented in the GraphPad web interface.

Supporting Information Figures

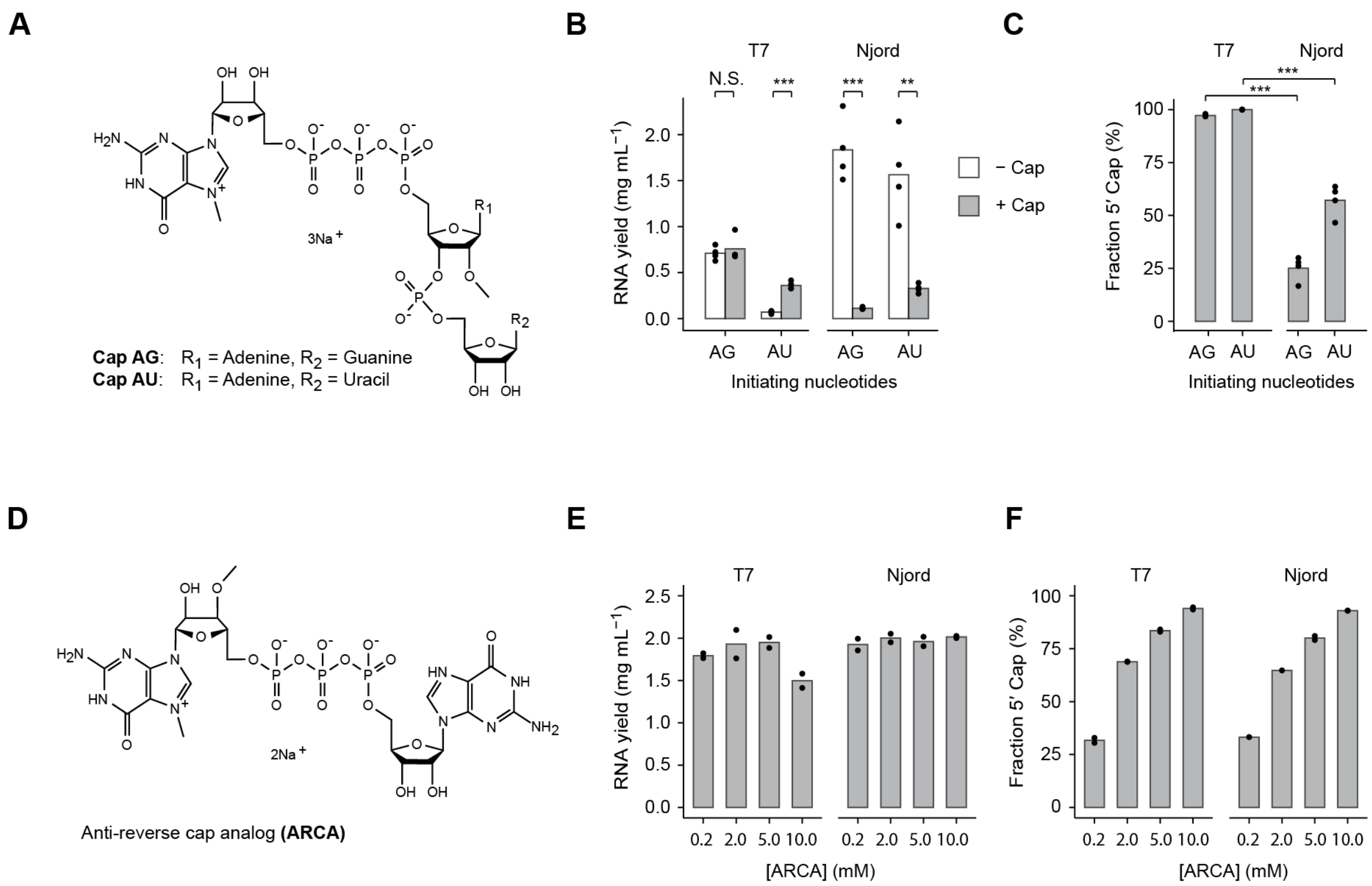

Figure S1. Incorporation of cap analogs using T7 or Njord RNAP. (**A**) The structures of trinucleotide cap analogs CapAG and CapAU. (**B**) RNA yields of saRNA with the initiating nucleotides AG or AU with or without cap analog included in the reaction. The relatively low RNA yields of T7 RNAP reflect the initially transcribed sequence used here (Table S8). Unlike T7 RNAP, Njord IVT reactions are poisoned by the inclusion of these trinucleotide cap analogs. (**C**) Incorporation of CapAG or CapAU using either T7 or Njord RNAP. T7 produces nearly completely capped mRNA, whereas Njord produces a mixture of capped and uncapped RNA species. (**D**) The structure of ARCA. (**E**) RNA yields of saRNA produced using either T7 or Njord RNAP at different concentrations of ARCA and 2 mM NTPs. (**F**) Effect of increasing ARCA concentration on the degree of cap incorporation. When ARCA is present at a 5-fold molar excess over GTP, over 90% of synthesized mRNA is capped using either polymerase. N.S., not significant ($p > 0.05$); *, $p < 0.05$; **, $p < 0.01$; ***, $p < 0.001$.

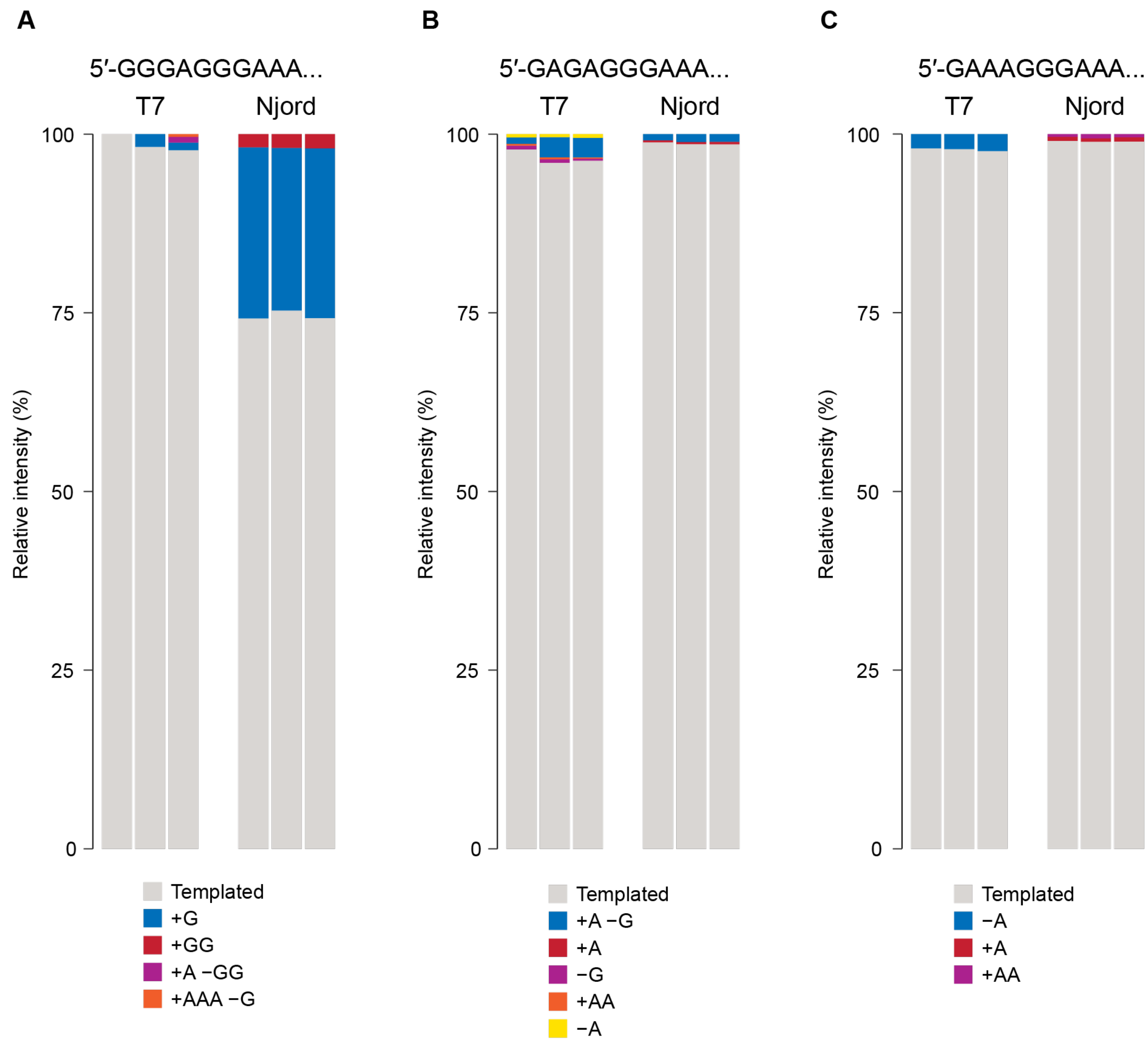

Figure S2. Comparison of 5′ homogeneity of RNA synthesized by T7 or Njord RNAPs with different initially transcribed sequences. Templates were prepared by PCR and encoded a transcript initiating with (**A**) GGG, (**B**) GAG or (**C**) GAA. Synthesized RNA (n = 3) was protected at the 5′ end with a biotinylated oligonucleotide, digested by RNase IV, purified and analyzed by UHPLC-MS/MS. Deconvoluted mass spectra were annotated according to the expected oligoribonucleotide and additions or deletions of purine residues. When the trinucleotide GGG is encoded at the 5′ end of the RNA, Njord has a greater propensity than T7 to incorporate additional untemplated guanosine residues. The 5′ homogeneity of RNA produced with Njord RNAP can be significantly improved by using GA as the initiating dinucleotide.

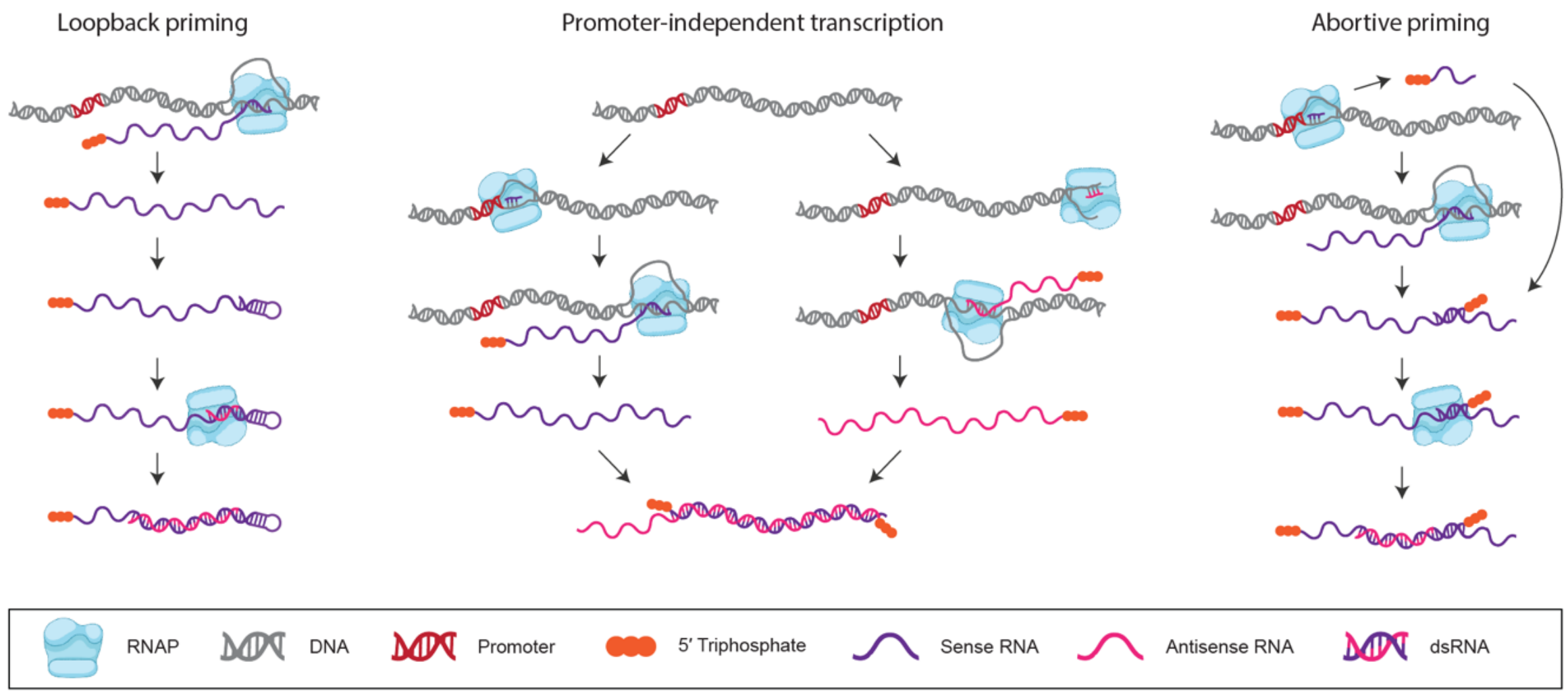

Figure S3. Routes for formation of different dsRNA species during IVT using RNA polymerase. Loopback priming refers to self-templated extension at the 3′ end of the transcript. Promoter-independent transcription describes an aberrant activity of T7 RNA polymerase in which DNA-dependent RNA polymerase activity is initiated at blunt ends of the dsDNA template. Finally, abortive priming reflects priming of the RNA-dependent RNA polymerase activity by short oligoribonucleotides produced as abortive transcripts.

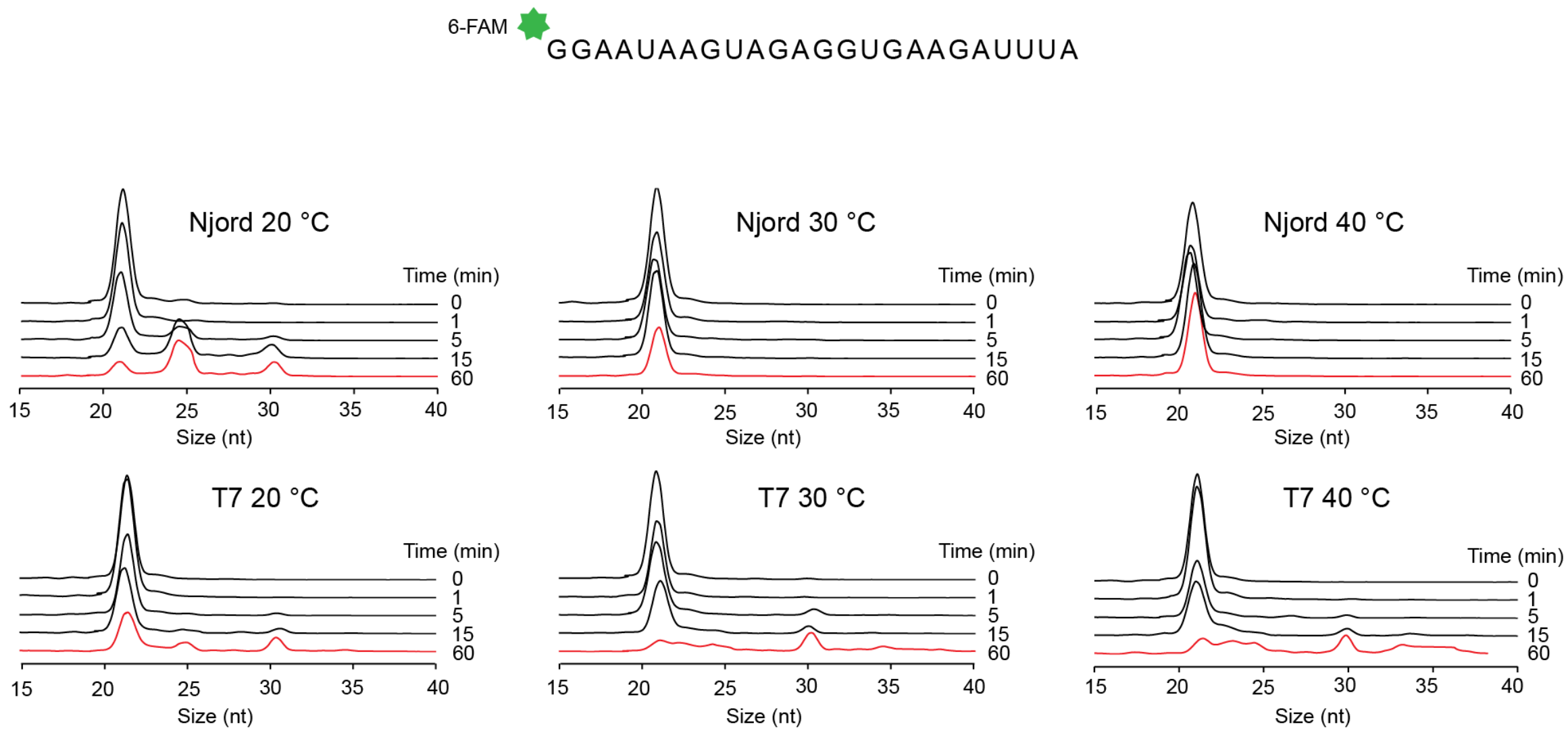

Figure S4. T7 and Njord RNAPs are both capable of RNA 3′ extension. At top is depicted the 5′-FAM labeled RNA substrate reacted with either polymerase. Below are capillary electrophoresis traces showing kinetics of extension of the oligoribonucleotide by either T7 or Njord RNAP at the indicated temperatures. Extended RNA species are apparent as shifted peaks in all of the reactions using T7 RNAP, and when Njord RNAP is used at 20 °C. For the T7 reactions, these peaks are known to correspond to self-templated extension resulting from RNA-dependent RNA polymerase activity (13). Similarity in migration of extended species produce using either polymerase at 20 °C demonstrate that Njord RNAP also possesses this activity.

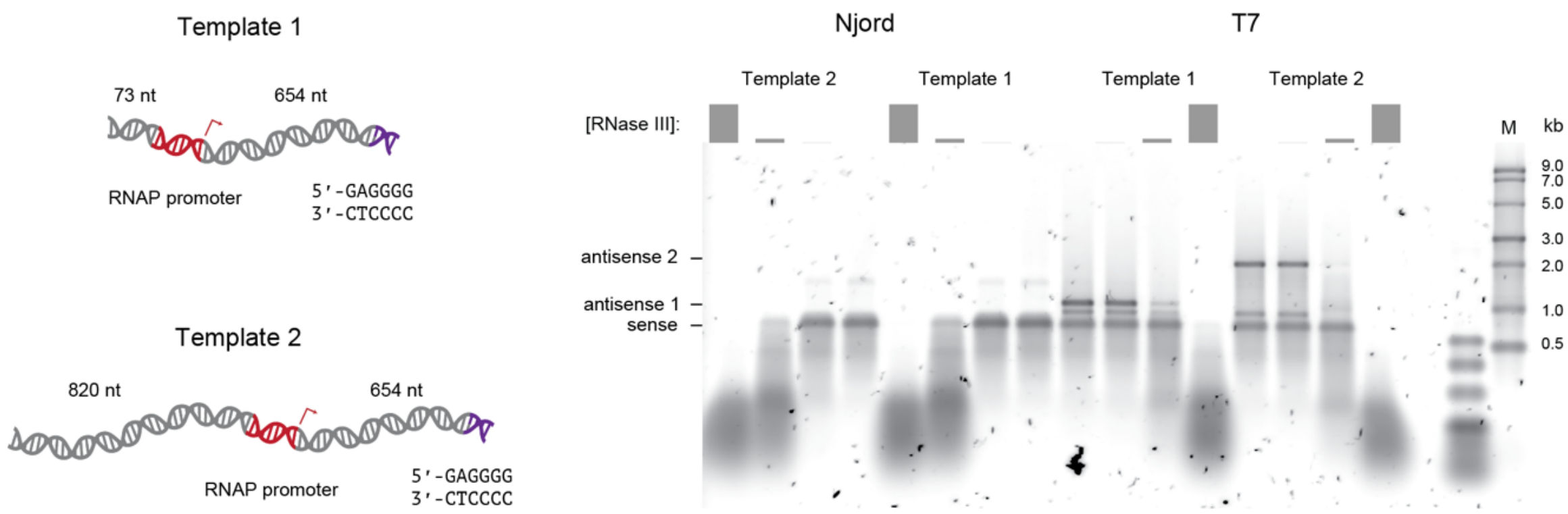

Figure S5. Promoter-independent transcription as a key differentiator of dsRNA formation by T7 and Njord RNAPs. On the left is a diagram of IVT templates used to assess promoter-independent transcription in Njord and T7 RNAP. These templates are distinct from the one used in Figure 3a although the terminal sequence is the same. On the right is shown an agarose gel of denatured RNA produced with promoter-independent transcription templates using Njord or T7 RNAPs. Antisense RNA species are identified by their gel shift and susceptibility to RNase III digestions.

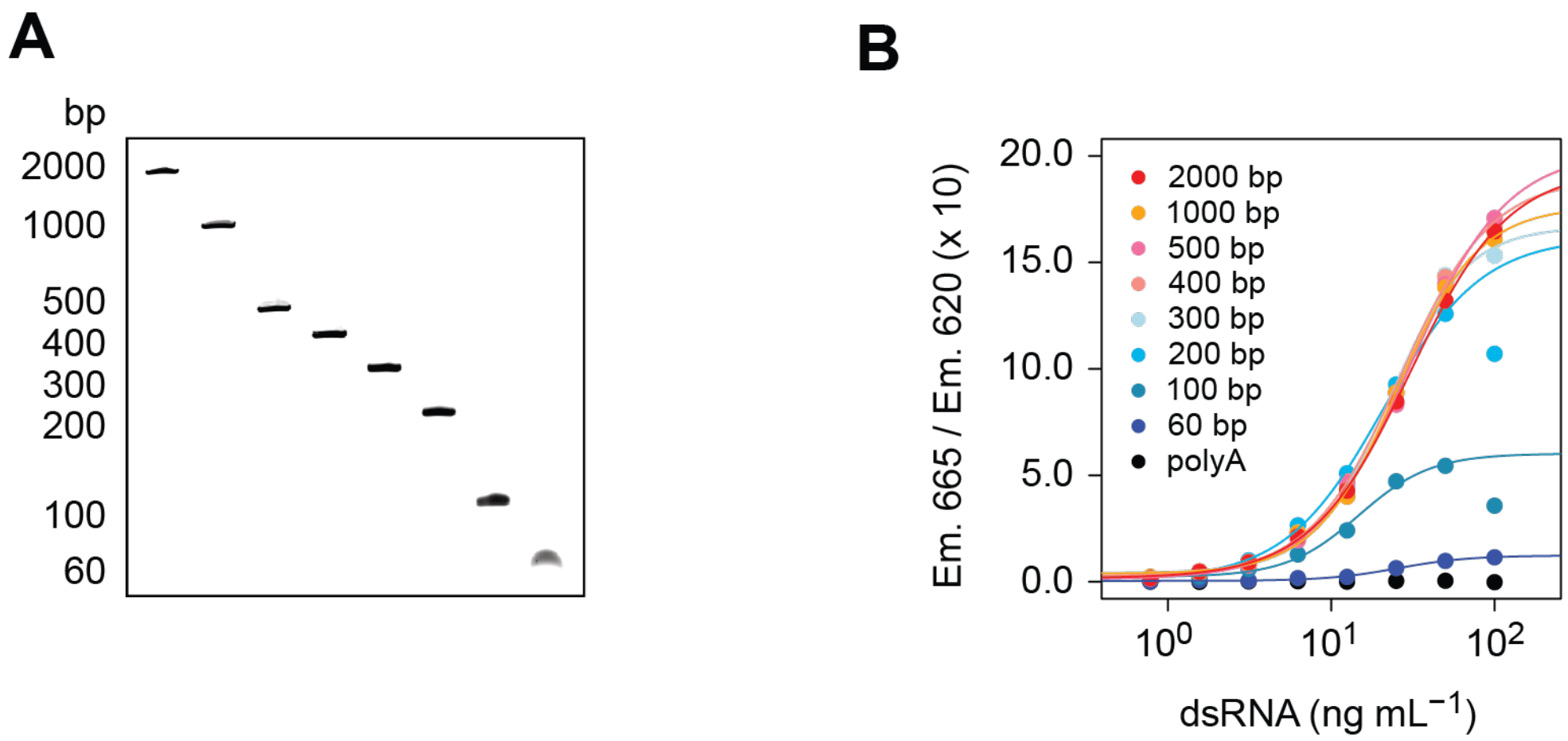

Figure S6. Preparation of dsRNA standards and their use in a homogenous time-resolved FRET assay. (**A**) Native polyacrylamide gel electrophoresis showing purified dsRNA standards. Standards were prepared by IVT of sense and anti-sense strands with Njord RNAP, annealing, and gel purification. (**B**) Response of the commercial HTRF assay to different concentrations of the dsRNA standards shows that the assay is accurate for larger dsRNA species. Shorter dsRNA species may be undetectable or difficult to quantify using this assay.

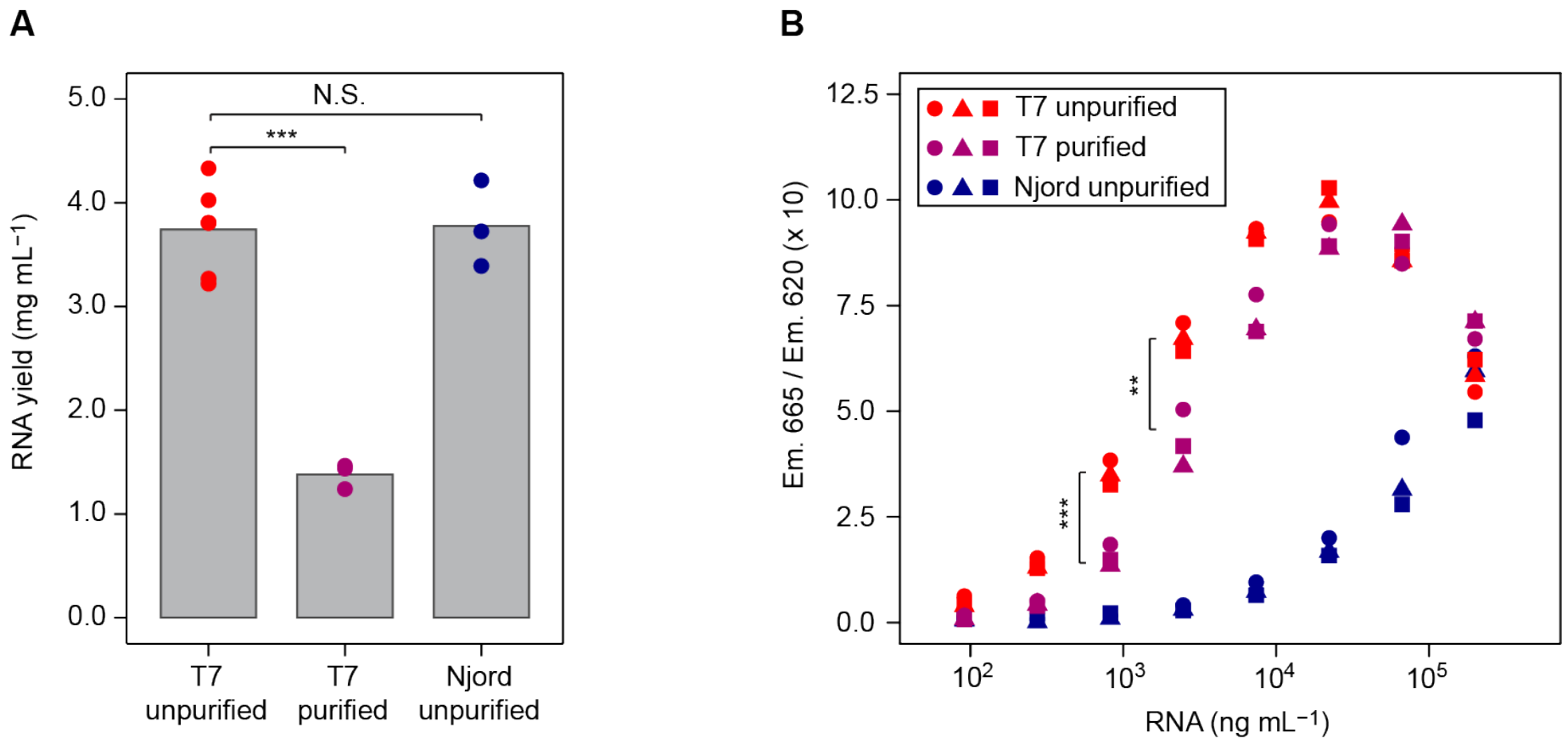

Figure S7. Comparison of T7-synthesized RNA purified by cellulose resin with Njord-synthesized RNA. (**A**) Yields of Fluc RNA synthesized by T7 RNAP before (n = 6) and after (n = 3) cellulose purification and by Njord RNAP (n = 3) without purification. T7 and Njord RNAPs produce comparable levels of RNA at their respective temperature optima, and cellulose purification incurs a loss in RNA yield. (**B**) HTRF measurement of dsRNA content in unpurified and purified T7-synthesized RNA compared to Njord-synthesized RNA. Batch cellulose purification reduces the dsRNA content of T7-synthesized RNA, but not to levels achieved with Njord RNAP. N.S., not significant ($p > 0.05$); *, $p < 0.05$; **, $p < 0.01$; ***, $p < 0.001$.

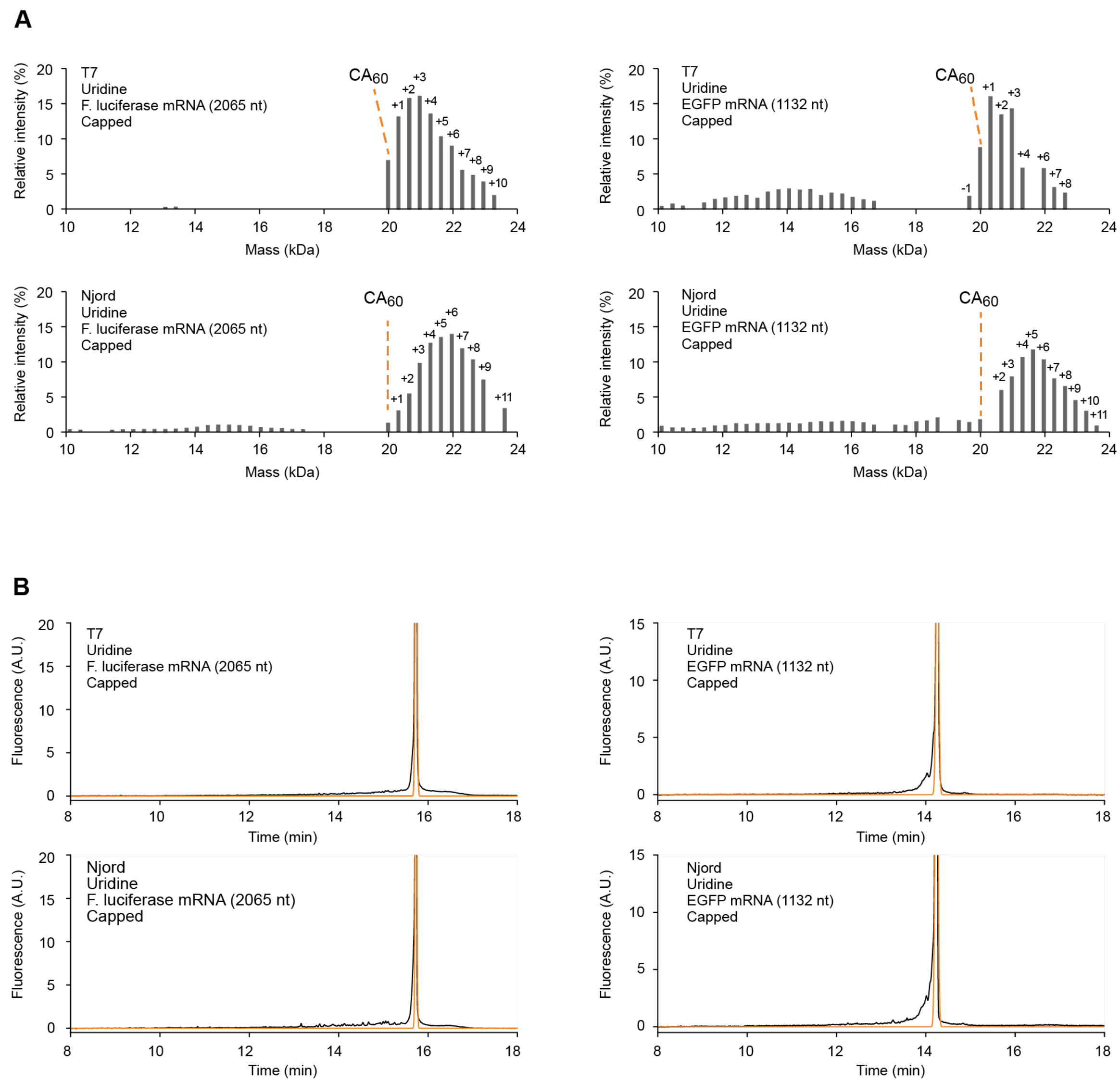

Figure S8. Analysis of mRNA 3′ tail homogeneity and RNA integrity. (**A**) Analysis of polyA tail length in EGFP and Fluc mRNAs prepared using either Njord or T7 RNAP. Digestion of the mRNA by RNase T1 and purification by oligodeoxythymidine beads is expected to yield a C(A_{60}) oligoribonucleotide corresponding to the polyA tail. Analysis of the purified digestions by UPLC-MS are shown. Species corresponding to oligoadenosine and C(A_n) with a 3′ cyclic phosphate were observed and attributed to off-target cleavage by RNase T1 but are not included here. Both T7 and Njord RNAPs extended the encoded polyA tail with additional adenosine residues (14). (**B**) Capillary gel electrophoresis analysis of EGFP and Fluc mRNAs prepared using either Njord or T7 RNAP. Orange curves represent a Gaussian function fit to the peak of interest. Pre-peak impurities have been previously ascribed to polyA tail heterogeneity and post-peak impurities to dsRNA (15).

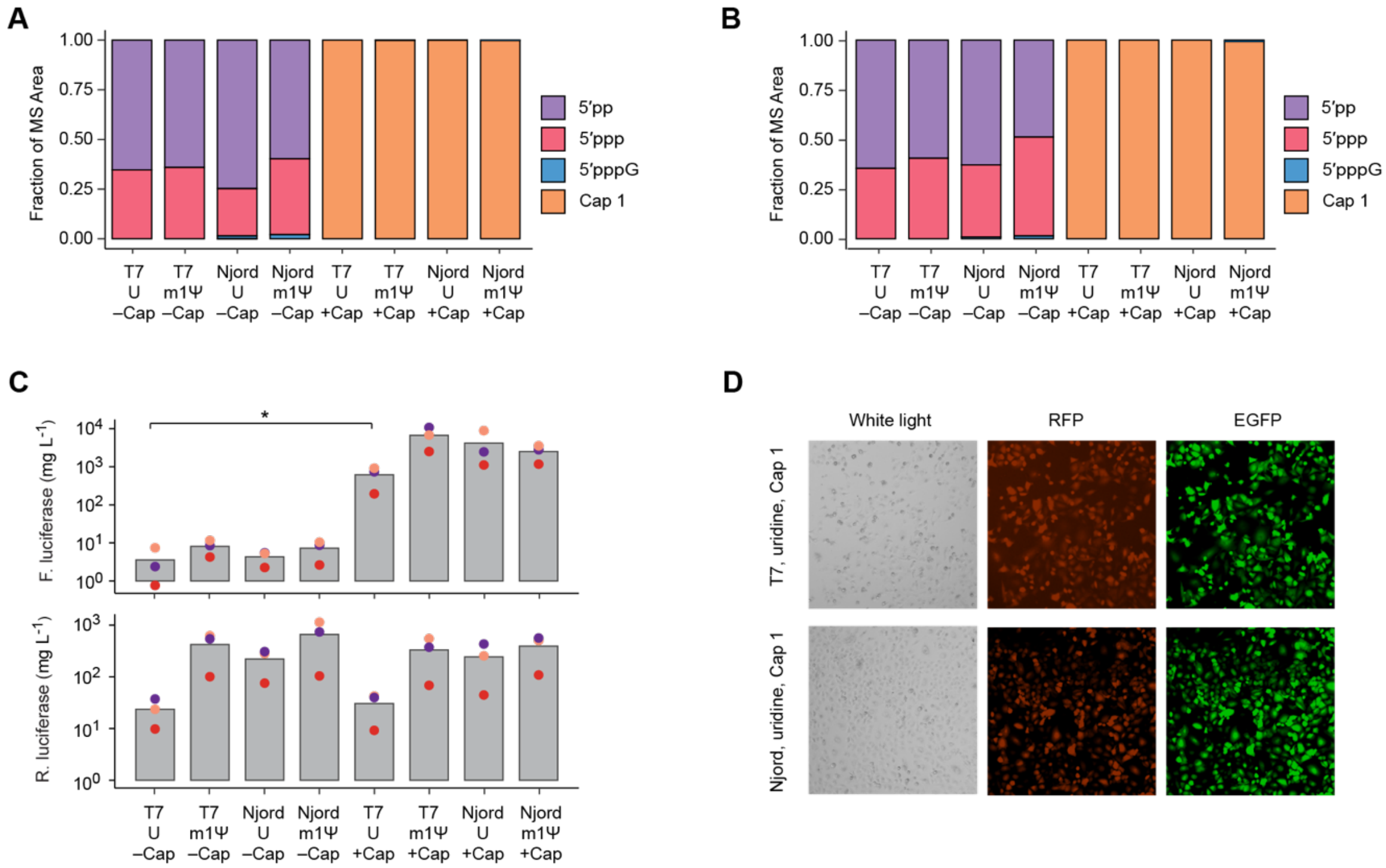

Figure S9. Cap-dependent translation of EGFP and Fluc mRNA. (**A**) Results of 5′ cap analysis by UHPLC-MS/MS for Fluc mRNA prepared under different conditions, before and after reaction with Faustovirus capping enzyme. (**B**) Results of 5′ cap analysis by UHPLC-MS/MS for EGFP mRNA. (**E**) Fluc and Rluc levels in A549 transfected with mRNA. Fluc mRNA (500 ng) prepared under different conditions was transfected alongside a control Rluc mRNA (50 ng) and expression levels were determined using a dual reporter assay. mRNA capping significantly enhanced the levels of Fluc expression. Unmodified dsRNA present in the T7 IVT products likely inhibits translation of the Rluc control mRNA. Data represent three biological replicates using the same mRNA preparations, and dots are colored according to the date of transfection. **(F)** Representative images of A549 cells expressing both experimental EGFP mRNA (500 ng) and control mCherry mRNA (50 ng). N.S., not significant ($p > 0.05$); *, $p < 0.05$; **, $p < 0.01$; ***, $p < 0.001$.

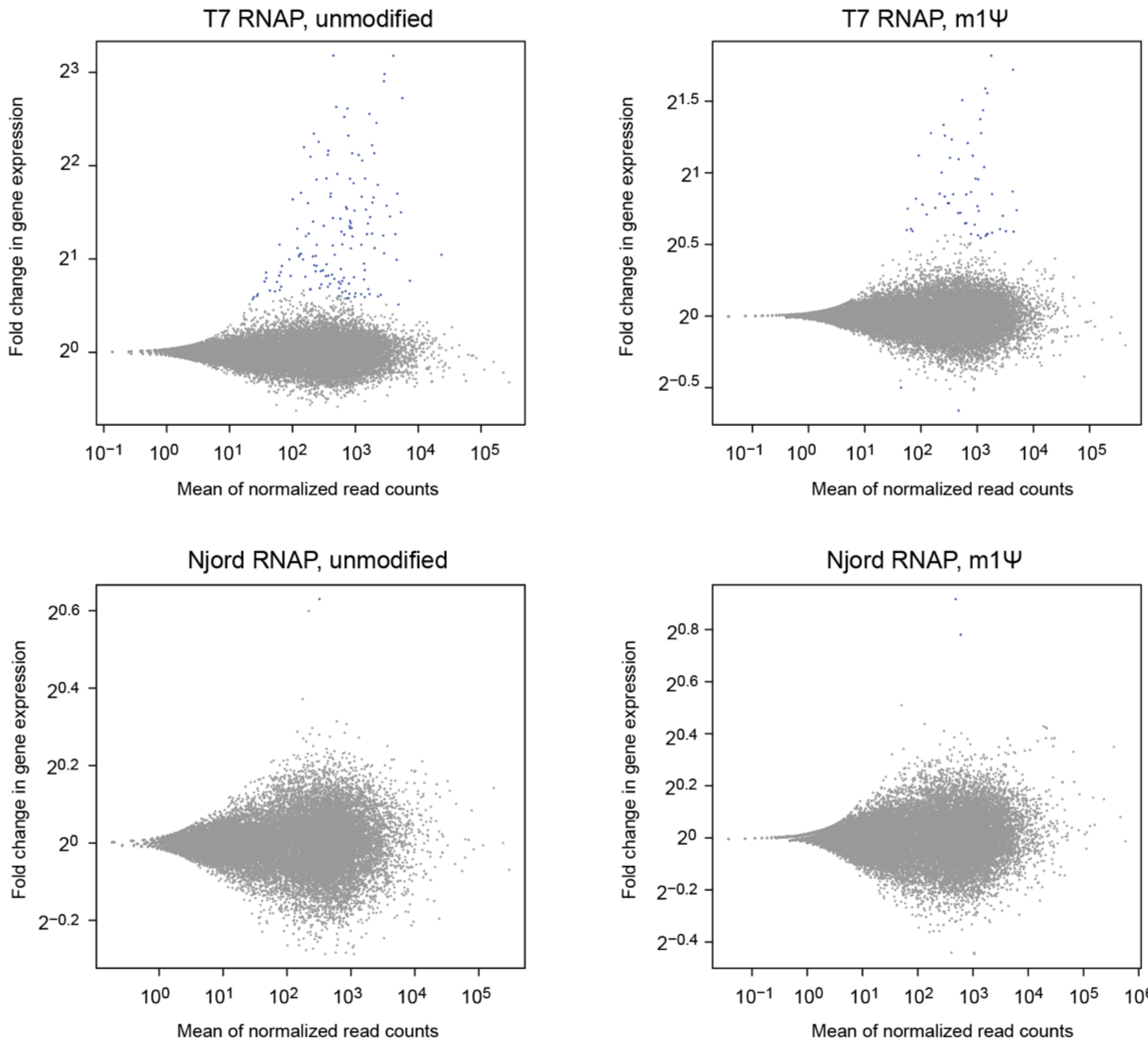

Figure S10. Plots of gene expression changes in A549 Dual cells in response to transfection with different mRNA species. Each dot represents a different gene. Dots colored blue are judged to be statistically significant (12) across two biological replicates.

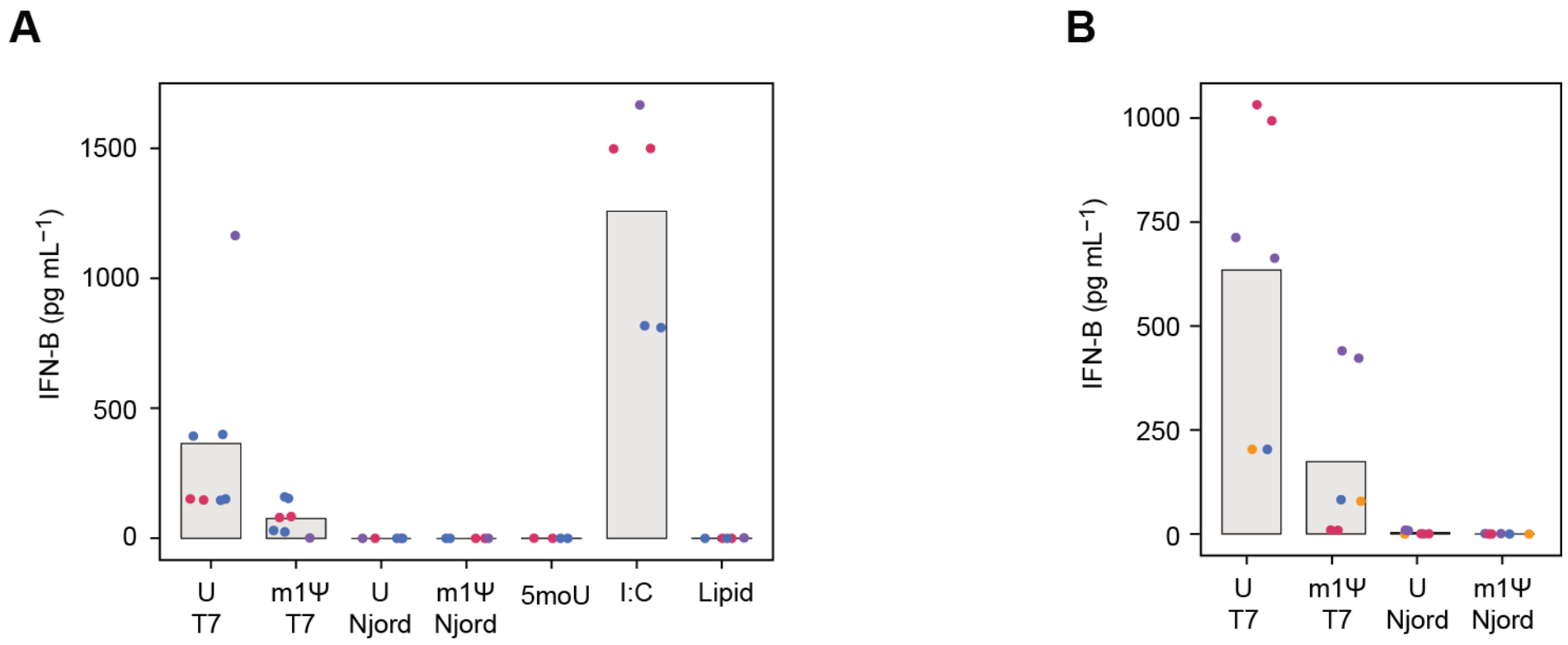

Figure S11. Secreted interferon-beta levels of A549 cells treated with capped Fluc (**A**) or EGFP (**B**) mRNA prepared using either T7 or Njord RNAP, with or without modified nucleotides. Dots are colored according to independent transfections using two different preparations of mRNA for each construct. 5moU, purified commercial 5-methoxyuridine Fluc; I:C, polyinosinic-polycytidylic acid; Lipid, transfection reagent prepared without RNA. Experiments for which the amount of secreted interferon are below the detection limit of the assay are indicated with points of value zero.

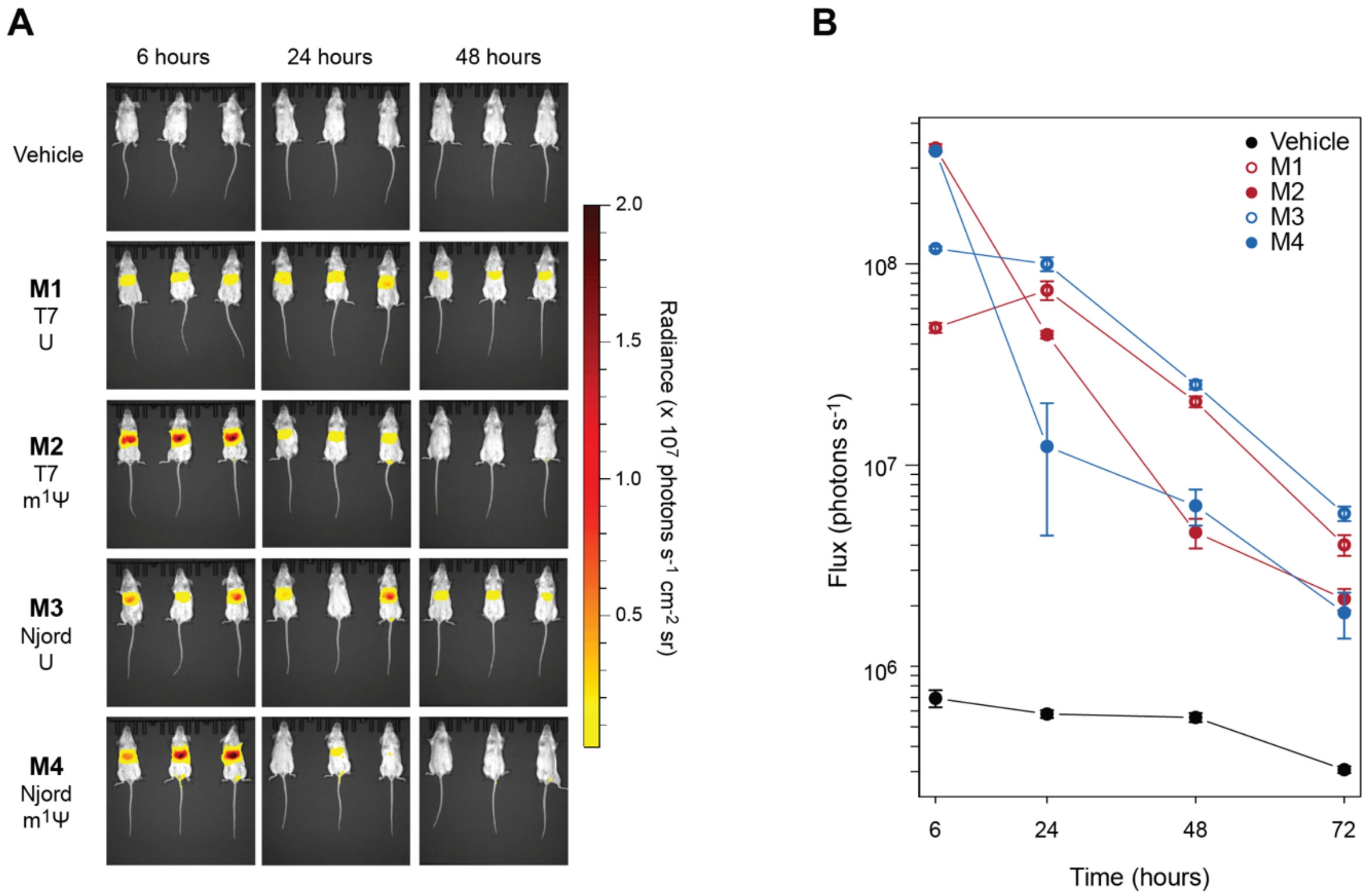

Figure S12. Kinetics of luciferase expression in mice treated with Fluc mRNA-LNPs. (**A**) In vivo images of luciferase expression in three mice treated with mRNA prepared using either T7 or Njord RNAP, with or without modified nucleotides. (**B**) Quantification of luciferase expression in these mouse model. Error bars represent standard error of the mean.

Supporting Information Tables

Table S1. Njord RNA polymerase promoters in the *Pseudomonas* phage Njord genome

ID	Promoter sequence[a]	Coordinate[b]
p1	ATTTAGGTTGCATTAGAGGCAAG	680
p2	GATTAGGTTGCATTATAGGAGAA	6314
p3	CTTTAGGTTGCAATATAGATAAG	9051
p4	GTTTAAGTTGCATTATAGATAAT	13791
p5	ATTTAAGTTGCATTATAGATAGA	15307
p6	TTTTAAGTTGCATTATAGGAGAA	21203
p7	AATTAAGTTGCATTATAGATAGG	24836
p8	GATTAAGTTGCATTAAAGGAGCT	35777
p9	ATTTAAGTTGCATTATAGGAGAG	43708

[a]Promoter sequences are given from position -17 to +6 according to the convention for T7 promoters. The initiating guanosine residue (+1) is underlined.

[b]Genome coordinate of the G(+1) residue of the promoter in the reference assembly for *Pseudomonas* phage Njord genome (GCF_003093955.1).

Table S2. Frequency of substitutions, insertions, and deletions during transcription by Njord and T7 RNA polymerases.

RNAP	Base[a]	Error frequency [b] (x 10^6 errors/base)	Substitution (%)	Insertion (%)	Deletion (%)	Sequenced bases
Njord	U	67 ± 8	80	6	14	45,041,484
	Ψ	105 ± 6	92	3	5	14,714,347
	$m^1\Psi$	118 ± 6	92	3	5	9,737,905
T7	U	51 ± 6	74	13	13	83,185,664
	Ψ	97 ± 3	91	4	5	28,603,208
	$m^1\Psi$	66 ± 3	89	6	5	13,863,557

[a]Uridine triphosphate included in the IVT reaction. U, uridine; Ψ, pseudouridine; $m^1\Psi$, *N*1-methylpseudouridine.

[b]Error frequencies are reported as an average and standard deviation over four experiments using two different RNA transcripts. These errors also reflect the fidelity of the reverse transcriptase and represent an upper bound for RNAP error rates.

Table S3. Frequency of specific nucleotide substitutions during transcription by Njord and T7 RNA polymerases.

RNAP	Base[a]	Frequency of substitution[b] (x 10^6 errors/base)											
		A|C	A|U	A|G	C|A	C|U	C|G	U|A	U|C	U|G	G|A	G|C	G|U
Njord	U	6	3	15	1	10	1	2	8	2	4	0	2
	Ψ	7	30	21	1	15	1	3	7	1	6	0	5
	m^1Ψ	10	13	38	2	12	3	6	10	2	9	0	3
T7	U	5	4	6	1	9	0	1	6	1	2	1	1
	Ψ	4	49	6	2	14	1	1	5	1	2	1	3
	m^1Ψ	6	10	9	5	9	1	3	7	2	5	1	1

[a]Uridine triphosphate included in the IVT reaction: U, uridine; Ψ, pseudouridine; m^1Ψ, *N*1-methylpseudouridine.

[b]Error frequencies are reported as an average over four experiments using two different RNA substrates. The notation A|C represents incorporation of a C residue when an A residue is expected in the RNA, corresponding to an rC:dT mismatch during transcription.

Table S4. Identification of 5′ triphosphate oligoribonucleotides in purified Fluc mRNA

Oligoribonucleotide	Strand	Position	Njord MS Area	T7 MS Area
GGGAAAUAAGA	Sense	G1-A11	7358266	2782273
GGGAAAUAAGAGA	Sense	G1-A13	319047	47424
GGGAAAUAAGAGAGA	Sense	G1-A15	141320	915
GGGAAAUAAGAGAGAA	Sense	G1-A16	83142	1399
GGGAAAUAAGAGAGAAA	Sense	G1-A17	574694	103809
GGGAAAUAAGAGAGAAAA	Sense	G1-A18	1151358	165292
GGGAAAUAAGAGAGAAAAGA	Sense	G1-A20	346821	45477
GGGAAAUAAGAGAGAAAAGAA	Sense	G1-A21	267261	36615
GGGAAAUAAGAGAGAAAAGAAGA	Sense	G1-A23	971385	17895
GGGAAAUAAG	Sense	G1-G10	665160	212861
GGGAAAUAAGAG	Sense	G1-G12	2907564	1100119
GGGAAAUAAGAGAG	Sense	G1-G14	495740	78849
GGGAAAUAAGAGAGAAAAG	Sense	G1-G19	655800	174517
GGGAAAUAAGAGAGAAAAGAAG	Sense	G1-G22	734995	4157
AUGAUGGGCAGCUUCUU	Antisense	A1569-U1585	77	255184
GGGCACCAGCAG	Antisense	G1139-G1150	8194	517888
GGGCACCAGCAGG	Antisense	G1139-G1151	2738	505313
GGGCACCAGCAGGG	Antisense	G1139-G1152	799	388217
GAACAUGCCGAAGCC	Antisense	G1256-C1270	512	223358
GGAUGAUGGGCAGCUUCUUC	Antisense	G1567-C1586	2649	696167
GGAUGAUGGG	Antisense	G1567-G1576	1166085	23808793
GGAUGAUGGGCAG	Antisense	G1567-G1579	25946	929650
GGAUGAUGGGCAGCUUCUUCUG	Antisense	G1567-G1588	1854	1190956
GGAUGAUGGGCAGCUU	Antisense	G1567-U1582	1104	3389379
GGAUGAUGGGCAGCUUCU	Antisense	G1567-U1584	3310	1990025
GGAUGAUGGGCAGCUUCUU	Antisense	G1567-U1585	3534	3055620
GAUGAUGGGCAGCUU	Antisense	G1568-U1582	317	6153284
GAUGAUGGGCAGCUUCU	Antisense	G1568-U1584	471	918450
GAUGAUGGGCAGCUUCUU	Antisense	G1568-U1585	856	5985949
GAUGGGCAGCUUCUUC	Antisense	G1571-C1586	528	823041
GAUGGGCAGCUUCUUCUGC	Antisense	G1571-C1589	356	501190
GAUGGGCAGCUUCUUCUG	Antisense	G1571-G1588	537	1774725
GAUGGGCAGCUUCUUCUGCACG	Antisense	G1571-G1592	1676	1630886
GAUGGGCAGCUU	Antisense	G1571-U1582	7006	342784
GAUGGGCAGCUUCU	Antisense	G1571-U1584	5549	1850838
GAUGGGCAGCUUCUU	Antisense	G1571-U1585	181	167368
GAUGGGCAGCUUCUUCU	Antisense	G1571-U1587	290	1055958
GAGUGGGGCAGG	Antisense	G239-G250	4724	433525
GGGGCAGGUGGAGG	Antisense	G243-G256	320	104641
GGAGGUGGGAG	Antisense	G252-G262	6756	590666

Table S5. Identification of 5′ triphosphate oligoribonucleotides in purified EGFP mRNA

Oligoribonucleotide	Strand	Position	Njord MS Area	T7 MS Area
AGUUUAACUAAGCUAUACUAACCCCAG	Sense	A1007-G1033	274755	8741
GGGAAAUAAGA	Sense	G1-A11	9731117	8841898
GGGAAAUAAGAGA	Sense	G1-A13	489580	235979
GGGAAAUAAGAGAGA	Sense	G1-A15	414892	117560
GGGAAAUAAGAGAGAA	Sense	G1-A16	168231	37894
GGGAAAUAAGAGAGAAA	Sense	G1-A17	2175098	559792
GGGAAAUAAGAGAGAAAA	Sense	G1-A18	1779954	659826
GGGAAAUAAGAGAGAAAAGA	Sense	G1-A20	879938	393909
GGGAAAUAAGAGAGAAAAGAA	Sense	G1-A21	807871	195044
GGGAAAUAAGAGAGAAAAGAAGA	Sense	G1-A23	2267190	140945
GGGAAAUAAGAGAGAAAAGAAGAGUAAGAAGAAAUAUAAGA	Sense	G1-A41	526819	53473156
GGGAAAUAA	Sense	G1-A9	938467	4599020
GGGAAAUAAG	Sense	G1-G10	1876500	961498
GGGAAAUAAGAG	Sense	G1-G12	6847418	3873855
GGGAAAUAAGAGAG	Sense	G1-G14	787348	121036
GGGAAAUAAGAGAGAAAAG	Sense	G1-G19	1086762	762046
GGGAAAUAAGAGAGAAAAGAAG	Sense	G1-G22	2468877	198837
AGCGGCUGAAGCA	Antisense	A850-A862	2037	60038
AGGGCACGGGCAGCUUGCCG	Antisense	A901-G920	42054	12978
GCUAAAGGUUAAU	Antisense	G134-U146	4884	725724
GAGUGGGGCAGG	Antisense	G239-G250	68883	1567396
GGGGCAGGUGGAGG	Antisense	G243-G256	1524	280590
GGAGGUGGGAG	Antisense	G252-G262	28839	1453969
GGUAGUGGU	Antisense	G469-U477	6987	389702
GGUUGUCGGGCAGCAGCACG	Antisense	G475-G494	737	345485
GGGCAGCAGCA	Antisense	G482-A492	378575	162738
GGUCGGCGAGCUGCACG	Antisense	G529-G545	1977	383571
GCGCUCCUGGACG	Antisense	G782-G794	4667	264673
GUGGUCGGGGUAG	Antisense	G839-G851	20919	711337
GGGGUAGCGGC	Antisense	G845-C855	755897	299189
GGCUGAAGCACUGCACG	Antisense	G853-G869	544	614491
GGCUGAAGCACU	Antisense	G853-U864	95266	2063818

Table S6. Description of plasmids used in this study.

Name[a]	Description	Figures
pNjord-01	Expression plasmid for Njord RNAP	1C
pNjord-02	Firefly luciferase IVT template for Njord RNAP	1D, 3B-D, 4A-C, 5A-C, S2, S7, S8, S9, S11, S12
pNjord-03	saRNA IVT template for Njord RNAP initiating with GG	1D, S1E-F
pNjord-04	*Cypridina* luciferase IVT template for Njord RNAP	2A, S5
pNjord-05	IVT template for Njord RNAP fidelity measurement	2B, 2C
pNjord-06	IVT template for Njord RNAP fidelity measurement	2B, 2C
pNjord-07	EGFP IVT template for Njord RNAP	3B, 3D, S8, S9, S11B
pNjord-08	Erythropoietin IVT template for Njord RNAP	3D
pNjord-09	saRNA IVT template for Njord RNAP initiating with AG	S1A-C
pNjord-10	saRNA IVT template for Njord RNAP initiating with AU	S1A-C
pT7-01	Firefly luciferase IVT template for T7 RNAP	1D, 3B-D, 4A-C, 5A-C, S2, S7, S8, S9, S11, S12
pT7-02	saRNA IVT template for T7 RNAP initiating with GG	1D, S1E-F, S6
pT7-03	*Cypridina* luciferase IVT template for T7 RNAP	1E, 2A, S5
pT7-04	IVT template for T7 RNAP fidelity measurement	2B, 2C
pT7-05	IVT template for T7 RNAP fidelity measurement	2B, 2C
pT7-06	PCR template for promoter independent transcription	3C
pT7-07	EGFP IVT template for T7 RNAP	3B, 3D, S8, S9, S11B
pT7-08	Erythropoietin IVT template for T7 RNAP	3D
pT7-09	saRNA IVT template for T7 RNAP initiating with AG	S1A-C
pT7-10	saRNA IVT template for T7 RNAP initiating with AU	S1A-C

Table S7. Sequences and descriptions of oligonucleotides used in this study.

Name[a]	Sequence[b]	Description
dOligo01	GCACAGAAAAGCATCTTACTTGGC	PCR primer with pT7-03 for Njord promoter mutations
dOligo02	TTTTAAGTTGCATTATAGGGAGACCCAAGCTTGGT	Forward PCR primer with pT7-03 for Njord promoter mutations
dOligo03	ATTTAAGTTGCATTATAGGGAGACCCAAGCTTGGT	Forward PCR primer with pT7-03 for Njord promoter mutations
dOligo04	CTTTAAGTTGCATTATAGGGAGACCCAAGCTTGGT	Forward PCR primer with pT7-03 for Njord promoter mutations
dOligo05	GTTTAAGTTGCATTATAGGGAGACCCAAGCTTGGT	Forward PCR primer with pT7-03 for Njord promoter mutations
dOligo06	TATTAAGTTGCATTATAGGGAGACCCAAGCTTGGT	Forward PCR primer with pT7-03 for Njord promoter mutations
dOligo07	TCTTAAGTTGCATTATAGGGAGACCCAAGCTTGGT	Forward PCR primer with pT7-03 for Njord promoter mutations
dOligo08	TGTTAAGTTGCATTATAGGGAGACCCAAGCTTGGT	Forward PCR primer with pT7-03 for Njord promoter mutations
dOligo09	TTATAAGTTGCATTATAGGGAGACCCAAGCTTGGT	Forward PCR primer with pT7-03 for Njord promoter mutations
dOligo10	TTCTAAGTTGCATTATAGGGAGACCCAAGCTTGGT	Forward PCR primer with pT7-03 for Njord promoter mutations
dOligo11	TTGTAAGTTGCATTATAGGGAGACCCAAGCTTGGT	Forward PCR primer with pT7-03 for Njord promoter mutations
dOligo12	TTTAAAGTTGCATTATAGGGAGACCCAAGCTTGGT	Forward PCR primer with pT7-03 for Njord promoter mutations
dOligo13	TTTCAAGTTGCATTATAGGGAGACCCAAGCTTGGT	Forward PCR primer with pT7-03 for Njord promoter mutations
dOligo14	TTTGAAGTTGCATTATAGGGAGACCCAAGCTTGGT	Forward PCR primer with pT7-03 for Njord promoter mutations
dOligo15	TTTTTAGTTGCATTATAGGGAGACCCAAGCTTGGT	Forward PCR primer with pT7-03 for Njord promoter mutations
dOligo16	TTTTCAGTTGCATTATAGGGAGACCCAAGCTTGGT	Forward PCR primer with pT7-03 for Njord promoter mutations
dOligo17	TTTTGAGTTGCATTATAGGGAGACCCAAGCTTGGT	Forward PCR primer with pT7-03 for Njord promoter mutations
dOligo18	TTTTATGTTGCATTATAGGGAGACCCAAGCTTGGT	Forward PCR primer with pT7-03 for Njord promoter mutations
dOligo19	TTTTACGTTGCATTATAGGGAGACCCAAGCTTGGT	Forward PCR primer with pT7-03 for Njord promoter mutations
dOligo20	TTTTAGGTTGCATTATAGGGAGACCCAAGCTTGGT	Forward PCR primer with pT7-03 for Njord promoter mutations
dOligo21	TTTTAAATTGCATTATAGGGAGACCCAAGCTTGGT	Forward PCR primer with pT7-03 for Njord promoter mutations
dOligo22	TTTTAATTTGCATTATAGGGAGACCCAAGCTTGGT	Forward PCR primer with pT7-03 for Njord promoter mutations
dOligo23	TTTTAACTTGCATTATAGGGAGACCCAAGCTTGGT	Forward PCR primer with pT7-03 for Njord promoter mutations
dOligo24	TTTTAAGATGCATTATAGGGAGACCCAAGCTTGGT	Forward PCR primer with pT7-03 for Njord promoter mutations
dOligo25	TTTTAAGCTGCATTATAGGGAGACCCAAGCTTGGT	Forward PCR primer with pT7-03 for Njord promoter mutations
dOligo26	TTTTAAGGTGCATTATAGGGAGACCCAAGCTTGGT	Forward PCR primer with pT7-03 for Njord promoter mutations
dOligo27	TTTTAAGTAGCATTATAGGGAGACCCAAGCTTGGT	Forward PCR primer with pT7-03 for Njord promoter mutations
dOligo28	TTTTAAGTCGCATTATAGGGAGACCCAAGCTTGGT	Forward PCR primer with pT7-03 for Njord promoter mutations
dOligo29	TTTTAAGTGGCATTATAGGGAGACCCAAGCTTGGT	Forward PCR primer with pT7-03 for Njord promoter mutations
dOligo30	TTTTAAGTTACATTATAGGGAGACCCAAGCTTGGT	Forward PCR primer with pT7-03 for Njord promoter mutations
dOligo31	TTTTAAGTTTCATTATAGGGAGACCCAAGCTTGGT	Forward PCR primer with pT7-03 for Njord promoter mutations
dOligo32	TTTTAAGTTCCATTATAGGGAGACCCAAGCTTGGT	Forward PCR primer with pT7-03 for Njord promoter mutations
dOligo33	TTTTAAGTTGAATTATAGGGAGACCCAAGCTTGGT	Forward PCR primer with pT7-03 for Njord promoter mutations
dOligo34	TTTTAAGTTGTATTATAGGGAGACCCAAGCTTGGT	Forward PCR primer with pT7-03 for Njord promoter mutations
dOligo35	TTTTAAGTTGGATTATAGGGAGACCCAAGCTTGGT	Forward PCR primer with pT7-03 for Njord promoter mutations
dOligo36	TTTTAAGTTGCTTTATAGGGAGACCCAAGCTTGGT	Forward PCR primer with pT7-03 for Njord promoter mutations
dOligo37	TTTTAAGTTGCCTTATAGGGAGACCCAAGCTTGGT	Forward PCR primer with pT7-03 for Njord promoter mutations
dOligo38	TTTTAAGTTGCGTTATAGGGAGACCCAAGCTTGGT	Forward PCR primer with pT7-03 for Njord promoter mutations
dOligo39	TTTTAAGTTGCAATATAGGGAGACCCAAGCTTGGT	Forward PCR primer with pT7-03 for Njord promoter mutations
dOligo40	TTTTAAGTTGCACTATAGGGAGACCCAAGCTTGGT	Forward PCR primer with pT7-03 for Njord promoter mutations
dOligo41	TTTTAAGTTGCAGTATAGGGAGACCCAAGCTTGGT	Forward PCR primer with pT7-03 for Njord promoter mutations
dOligo42	TTTTAAGTTGCATAATAGGGAGACCCAAGCTTGGT	Forward PCR primer with pT7-03 for Njord promoter mutations
dOligo43	TTTTAAGTTGCATCATAGGGAGACCCAAGCTTGGT	Forward PCR primer with pT7-03 for Njord promoter mutations
dOligo44	TTTTAAGTTGCATGATAGGGAGACCCAAGCTTGGT	Forward PCR primer with pT7-03 for Njord promoter mutations
dOligo45	TTTTAAGTTGCATTTTAGGGAGACCCAAGCTTGGT	Forward PCR primer with pT7-03 for Njord promoter mutations
dOligo46	TTTTAAGTTGCATTCTAGGGAGACCCAAGCTTGGT	Forward PCR primer with pT7-03 for Njord promoter mutations
dOligo47	TTTTAAGTTGCATTGTAGGGAGACCCAAGCTTGGT	Forward PCR primer with pT7-03 for Njord promoter mutations
dOligo48	TTTTAAGTTGCATTAAAGGGAGACCCAAGCTTGGT	Forward PCR primer with pT7-03 for Njord promoter mutations
dOligo49	TTTTAAGTTGCATTACAGGGAGACCCAAGCTTGGT	Forward PCR primer with pT7-03 for Njord promoter mutations
dOligo50	TTTTAAGTTGCATTAGAGGGAGACCCAAGCTTGGT	Forward PCR primer with pT7-03 for Njord promoter mutations
dOligo51	TTTTAAGTTGCATTATTGGGAGACCCAAGCTTGGT	Forward PCR primer with pT7-03 for Njord promoter mutations
dOligo52	TTTTAAGTTGCATTATCGGGAGACCCAAGCTTGGT	Forward PCR primer with pT7-03 for Njord promoter mutations
dOligo53	TTTTAAGTTGCATTATGGGGAGACCCAAGCTTGGT	Forward PCR primer with pT7-03 for Njord promoter mutations
dOligo54	TTTTAAGTTGCATTATAAGGAGACCCAAGCTTGGT	Forward PCR primer with pT7-03 for Njord promoter mutations
dOligo55	TTTTAAGTTGCATTATATGGAGACCCAAGCTTGGT	Forward PCR primer with pT7-03 for Njord promoter mutations
dOligo56	TTTTAAGTTGCATTATACGGAGACCCAAGCTTGGT	Forward PCR primer with pT7-03 for Njord promoter mutations
dOligo57	TTTTAAGTTGCATTATAGAGAGACCCAAGCTTGGT	Forward PCR primer with pT7-03 for Njord promoter mutations
dOligo58	TTTTAAGTTGCATTATAGTGAGACCCAAGCTTGGT	Forward PCR primer with pT7-03 for Njord promoter mutations
dOligo59	TTTTAAGTTGCATTATAGCGAGACCCAAGCTTGGT	Forward PCR primer with pT7-03 for Njord promoter mutations
dOligo60	TACAGTTCACGAGGACCGTCAAGA	Primer for first strand cDNA synthesis in fidelity assay
dOligo61	AGAGTACACGAGTCAGGCTACAGCATC	Primer for second strand cDNA synthesis in fidelity assay
dOligo62	CCCCTCAGTTGTACAGTTCATCCATGCCATCCCAGGATTACCATGTACGC	Reverse PCR primer with pT7-06 for IVT templates prone to dsRNA
dOligo63	AAATTTAAATTTAAATAATACGACTCACTATAGGCTCGTTATAGCCAACATAACTGCG	Forward PCR primer with pT7-06 for T7 IVT template
dOligo64	AAATTTAAATTTAAAGTTTAAGTTGCATTATAGGCTCGTTATAGCCAACATAACTGCG	Forward PCR primer with pT7-06 with Njord IVT template
dOligo65	GTACGGTGGGAGGTCTATATAAGC	Forward PCR primer with pT7-03 or pNjord-04 for shorter IVT template
dOligo66	AGTACAATCTGCTCTGATGCCG	Forward PCR primer with pT7-03 or pNjord-04 for longer IVT template
dOligo67	CCCCTCAGTTGTACAGTTCATCCATGCCATGGCGATTCTTACAGATCTTCCTCC	Reverse PCR primer with pT7-03 or pNjord-04 for IVT templates
dOligo68	GGTGAGTTGCGAAGGCTAC	Forward PCR primer with pT7-02 for IVT template for top dsRNA strand
dOligo69	AAAAAGTTTAAGTTGCATTATAGAAGCCCTCGCTATTGTGTGTAAC	Reverse PCR primer with pT7-02 for IVT template for dsRNA strand (100nt)
dOligo70	AAAAAGTTTAAGTTGCATTATAGGCCATTATACCAGTCATCTGATCG	Reverse PCR primer with pT7-02 for IVT template for dsRNA strand (200nt)
dOligo71	AAAAAGTTTAAGTTGCATTATAGGTGTTGGTGTTCCTGTTAGTCC	Reverse PCR primer with pT7-02 for IVT template for dsRNA strand (300nt)
dOligo72	AAAAAGTTTAAGTTGCATTATAGTTCTCTAGTACCCAGCATTTTCTCG	Reverse PCR primer with pT7-02 for IVT template for dsRNA strand (400nt)
dOligo73	AAAAAGTTTAAGTTGCATTATAGAGGCTGGGACTTTTACGCAG	Reverse PCR primer with pT7-02 for IVT template for dsRNA strand (500nt)
dOligo74	AAAAAGTTTAAGTTGCATTATAGGCATCAGTACTTTAGCGTCGTATG	Reverse PCR primer with pT7-02 for IVT template for dsRNA strand (1000nt)
dOligo75	AAAAAGTTTAAGTTGCATTATAGGACGGCATACACTCCTTTTC	Reverse PCR primer with pT7-02 for IVT template for dsRNA strand (2000nt)
dOligo76	AAAAAGTTTAAGTTGCATTATAGGTGAGTTGCGAAGGCTAC	Forward PCR primer with pT7-02 for IVT template for bottom dsRNA strand
dOligo77	GAAGCCCTCGCTATTGTGTGTAAC	Reverse PCR primer with pT7-02 for IVT template for dsRNA strand (100nt)
dOligo78	GGCCATTATACCAGTCATCTGATCG	Reverse PCR primer with pT7-02 for IVT template for dsRNA strand (200nt)
dOligo79	GGTGTTGGTGTTCCTGTTAGTCC	Reverse PCR primer with pT7-02 for IVT template for dsRNA strand (300nt)
dOligo80	GTTCTCTAGTACCCAGCATTTTCTCG	Reverse PCR primer with pT7-02 for IVT template for dsRNA strand (400nt)
dOligo81	GAGGCTGGGACTTTTACGCAG	Reverse PCR primer with pT7-02 for IVT template for dsRNA strand (500nt)
dOligo82	GGCATCAGTACTTTAGCGTCGTATG	Reverse PCR primer with pT7-02 for IVT template for dsRNA strand (1000nt)
dOligo83	GGACGGCATACACTCCTTTTC	Reverse PCR primer with pT7-02 for IVT template for dsRNA strand (2000nt)
dOligo84	CACGATCTCCTTCTCGG	Reverse PCR primer for 5′ homogeneity IVT templates
dOligo85	TATAATACGACTCACTATAGGGAGGGAAATAAGAGAGAAAAGAAGAGTAAG	Forward PCR primer for T7 GGG 5′ homogeneity IVT template
dOligo86	TATAATACGACTCACTATAGAGAGGGAAATAAGAGAGAAAAGAAGAGTAAG	Forward PCR primer for T7 GAG 5′ homogeneity IVT template
dOligo87	TATAATACGACTCACTATAGAAAGGGAAATAAGAGAGAAAAGAAGAGTAAG	Forward PCR primer for T7 GAA 5′ homogeneity IVT template
dOligo88	TAATTTAAGTTGCATTATAGGGAGGGAAATAAGAGAGAAAAGAAGAGTAAG	Forward PCR primer for Njord GGG 5′ homogeneity IVT template
dOligo89	TAATTTAAGTTGCATTATAGAGAGGGAAATAAGAGAGAAAAGAAGAGTAAG	Forward PCR primer for Njord GAG 5′ homogeneity IVT template
dOligo90	TAATTTAAGTTGCATTATAGAAAGGGAAATAAGAGAGAAAAGAAGAGTAAG	Forward PCR primer for Njord GAA 5′ homogeneity IVT template
dOligo91	/5BiosG/GTGTGTACTACGCCGTCAATCT	Protecting oligo for analysis of 5′ CapAG incorporation
dOligo92	/5BiosG/GTGTGTACTACGCCGTCAATAT	Protecting oligo for analysis of 5′ CapAU incorporation
dOligo93	/5BiosG/GTGTGTACTACGCCGTCAATCC	Protecting oligo for analysis of 5′ ARCA incorporation
dOligo94	CTTCTTTTCTCTCTTATTTCCC/3deSBioTEG/	Protecting oligo for 5′ enzymatic capping and 5′ homogeneity MS analysis
rOligo01	/56-FAM/GGAAUAAGUAGAGGUGAAGAUUUA	Labeled RNA for capillary electrophoresis assay
rOligo02	GACGACCUGCAAAGUCUAUUCAACACGGGUCUCAAAACAGGAGAGCGACAGCGCUUCAAA	Top strand for dsRNA 60nt standard
rOligo03	UUUGAAGCGCUGUCGCUCUCCUGUUUUGAGACCCGUGUUGAAUAGACUUUGCAGGUCGUC	Bottom strand for dsRNA 60 nt standard

[a]dOligo denotes an oligodeoxyribonucleotide and rOligo denotes an oligoribonucleotide.
[b]/BiosG/ denotes a 5′ biotin modification, /3deSBioTEG/ denotes a 3′ desthiobiotin-TEG modification and /56-FAM/ denotes a 5′ fluorescein modification.

Table S8. Description of RNA species synthesized in this study.

Name	RNAP	Length (nt)	GC content (%)	ITS[a]	Templates[b]	PCR Primers[c]	Figures
Fluc	T7, Njord	2065	61.6	GGGAAAUAAGAGAGA	pT7-01, pNjord-02	N/A	1D, 3B-D, 4A-C, 5A-C, S7, S8, S9, S11A, S12
EGFP	T7, Njord	1132	56.3	GGGAAAUAAGAGAGA	pT7-07, pNjord-07	N/A	3B, 3D, S8, S9, S11B
EPO	T7, Njord	994	61.2	GGGAAAUAAGAGAGA	pT7-08, pNjord-08	N/A	3D
saRNA-GG	T7, Njord	9707	49.5	GGAUUGACGGCGUAG	pT7-02, pNjord-03	N/A	1D, S1E-F
saRNA-AG	T7, Njord	9707	49.5	AGAUUGACGGCGUAG	pT7-09, pNjord-09	N/A	S1E-F
saRNA-AU	T7, Njord	9707	49.5	AUAUUGACGGCGUAG	pT7-10, pNjord-10	N/A	S1E-F
Cluc	T7, Njord	1707	48.9	GGAGAACCCAAGCUU	pT7-03, pNjord-04	N/A	2A
Fidelity-1	T7, Njord	1158	49.7	GGGUCUAGAAAUAAU	pT7-04, pNjord-05	N/A	2B, 2C
Fidelity-2	T7, Njord	1160	49.6	GGGUCUAGAAAUAAU	pT7-05, pNjord-06	N/A	2B, 2C
PromoterMod	Njord	1809	47.9	NNGGAGAACCCAAGC	pT7-03	dOligo01-59	1E
dsRNA-1	T7, Njord	343	49.3	GGCUCGUUAUAGCCA	pT7-06	dOligo62-64	3A
dsRNA-2	T7, Njord	654	49.7	GGAGAACCCAAGCUU	pT7-03, pNjord-04	dOligo65-67	S5
Sense-100nt	Njord	100	54	GGUGAGUUGCGAAGG	pT7-02	dOligo76,77	S6
Sense-200nt	Njord	205	51.2	GGUGAGUUGCGAAGG	pT7-02	dOligo76,78	S6
Sense-300nt	Njord	298	49.7	GGUGAGUUGCGAAGG	pT7-02	dOligo76,79	S6
Sense-400nt	Njord	401	48.4	GGUGAGUUGCGAAGG	pT7-02	dOligo76,80	S6
Sense-500nt	Njord	501	49.8	GGUGAGUUGCGAAGG	pT7-02	dOligo76,81	S6
Sense-1000nt	Njord	996	50.8	GGUGAGUUGCGAAGG	pT7-02	dOligo76,82	S6
Sense-2000nt	Njord	1970	50.5	GGUGAGUUGCGAAGG	pT7-02	dOligo76,83	S6
Antisense-200nt	Njord	205	51.2	GGCCAUUAUACCAGU	pT7-02	dOligo68,70	S6
Antisense-300nt	Njord	298	49.7	GGUGUUGGUGUUCCU	pT7-02	dOligo68,71	S6
Antisense-400nt	Njord	401	48.4	GUUCUCUAGUACCCA	pT7-02	dOligo68.72	S6
Antisense-500nt	Njord	501	49.8	GAGGCUGGGACUUUU	pT7-02	dOligo68,73	S6
Antisense-1000nt	Njord	996	50.8	GGCAUCAGUACUUUA	pT7-02	dOligo68,74	S6
Antisense-2000nt	Njord	1970	50.5	GGACGGCAUACACUC	pT7-02	dOligo68,75	S6
HomogeneityGGG	T7, Njord	1558	64.6	GGGAGGGAAATAAGA	pT7-01, pNjord-02	dOligo84,85,88	S2
HomogeneityGAG	T7, Njord	1558	64.5	GAGAGGGAAATAAGA	pT7-01, pNjord-02	dOligo84,86,89	S2
HomogeneityGAA	T7, Njord	1558	64.5	GAAAGGGAAATAAGA	pT7-01, pNjord-02	dOligo84,87,90	S2

[a]Initially transcribed sequence.
[b]Annotated PCR and IVT template sequences are provided as an accompanying GenBank file.
[c]PCR primers are given for IVT templates prepared by PCR. Sequences are available in Table S7.

Supporting Information References

1. N. A. O'Leary, *et al.*, Reference sequence (RefSeq) database at NCBI: current status, taxonomic expansion, and functional annotation. *Nucleic Acids Research* **44**, D733–D745 (2016).

2. D. Turner, *et al.*, Summary of taxonomy changes ratified by the International Committee on Taxonomy of Viruses (ICTV) from the Bacterial Viruses Subcommittee, 2025. *J Gen Virol* **106** (2025).

3. J. P. Meier-Kolthoff, M. Göker, VICTOR: genome-based phylogeny and classification of prokaryotic viruses. *Bioinformatics* **33**, 3396–3404 (2017).

4. G. Ambrosini, R. Groux, P. Bucher, PWMScan: a fast tool for scanning entire genomes with a position-specific weight matrix. *Bioinformatics* **34**, 2483–2484 (2018).

5. T.-H. Chen, V. Potapov, N. Dai, J. L. Ong, B. Roy, N1-methyl-pseudouridine is incorporated with higher fidelity than pseudouridine in synthetic RNAs. *Scientific Reports* **12**, 13017 (2022).

6. L. Greenough, *et al.*, Adapting capillary gel electrophoresis as a sensitive, high-throughput method to accelerate characterization of nucleic acid metabolic enzymes. *Nucleic Acids Research* **44**, e15–e15 (2016).

7. S. R. Strezsak, P. J. Beuning, N. J. Skizim, Complete enzymatic digestion of double-stranded RNA to nucleosides enables accurate quantification of dsRNA. *Analytical Methods* **13**, 179–185 (2021).

8. E. J. Wolf, N. Dai, S.-H. Chan, I. R. Jr. Corrêa, Selective characterization of mRNA 5′ end-capping by DNA probe-directed enrichment with site-specific endoribonucleases. *ACS Pharmacol. Transl. Sci.* **6**, 1692–1702 (2023).

9. A. Gordon, Fastx-toolkit. *FASTQ/A short-reads preprocessing tools.* **5** (2010).

10. A. Dobin, *et al.*, STAR: ultrafast universal RNA-seq aligner. *Bioinformatics* **29**, 15–21 (2013).

11. Y. Liao, G. K. Smyth, W. Shi, featureCounts: an efficient general purpose program for assigning sequence reads to genomic features. *Bioinformatics* **30**, 923–930 (2014).

12. M. I. Love, W. Huber, S. Anders, Moderated estimation of fold change and dispersion for RNA-seq data with DESeq2. *Genome Biology* **15**, 550 (2014).

13. Y. Gholamalipour, A. Karunanayake Mudiyanselage, Craig T Martin, 3′ end additions by T7 RNA polymerase are RNA self-templated, distributive and diverse in character—RNA-Seq analyses. *Nucleic Acids Research* **46**, 9253–9263 (2018).

14. L. E. Macdonald, Y. Zhou, W. T. McAllister, Termination and slippage by bacteriophage T7 RNA polymerase. *J. Mol. Biol.* **232**, 1030-1047 (1993).

15. J. Camperi, et al., Comprehensive impurity profiling of mRNA: Evaluating current technologies and advanced analytical techniques. *Anal. Chem.* **96**, 3886–3897 (2024).
